# Exposure to heavy metals and red blood cell parameters in children: A systematic review of observational studies

**DOI:** 10.3389/fped.2022.921239

**Published:** 2022-10-06

**Authors:** Carolina Capitão, Raquel Martins, Osvaldo Santos, Manuel Bicho, Tamás Szigeti, Andromachi Katsonouri, Beatrice Bocca, Flavia Ruggieri, Wojciech Wasowicz, Hanna Tolonen, Ana Virgolino

**Affiliations:** ^1^Environmental Health Behaviour Lab, Instituto de Saúde Ambiental, Faculdade de Medicina, Universidade de Lisboa, Lisbon, Portugal; ^2^Laboratório Associado TERRA, Faculdade de Medicina, Universidade de Lisboa, Lisbon, Portugal; ^3^Unbreakable Idea Research, Cadaval, Portugal; ^4^Laboratório de Genética, Instituto de Saúde Ambiental, Faculdade de Medicina, Universidade de Lisboa, Lisbon, Portugal; ^5^Instituto Rocha Cabral, Lisbon, Portugal; ^6^National Public Health Center, Budapest, Hungary; ^7^State General Laboratory, Ministry of Health, Nicosia, Cyprus; ^8^Department of Environment and Health, Istituto Superiore di Sanità, Rome, Italy; ^9^Nofer Institute of Occupational Medicine, Lodz, Poland; ^10^Department of Public Health and Welfare, Finnish Institute for Health and Welfare (THL), Helsinki, Finland

**Keywords:** HBM4EU, biomonitoring, hematology, erythrocyte indices, child

## Abstract

**Background:**

Mechanistic studies show that heavy metals interfere with the hematopoietic system by inhibiting key enzymes, which could lead to anemia. However, the link between children's exposure and red blood cell (RBC) parameters has been inconsistent. We aimed to summarize evidence on human studies exploring the association between exposure to lead, mercury, cadmium, arsenic, and chromium VI and RBC parameters in children.

**Methods:**

Following the PRISMA guidelines, we searched PubMed, Scopus, and Web of Science databases for studies published between January 2010 and April 2022. Eligible papers included human observational studies that directly assessed exposure (internal dose) to the heavy metals under study and RBC parameters in participants aged ≤ 18 years. We excluded studies using hospital-based samples. Study quality was assessed using the National Institutes of Health's Quality Assessment Tools for Cohort and Cross-Sectional Studies. We synthesized the evidence using vote counting based on the direction of the relationship.

**Results:**

Out of 6,652 retrieved papers, we included a total of 38 (33 assessing lead, four mercury, two cadmium, and two arsenic; chromium VI was not assessed in any included paper). More than half of the studies were conducted in Asia. We found evidence of a positive relationship between lead concentration and hemoglobin (proportion of studies reporting negative relationships = 0.750; 95% Confidence Interval (CI) 0.583, 0.874) and mean corpuscular hemoglobin (0.875; 95% CI 0.546, 0.986), and a positive relationship with red cell distribution width (0.000; 95%CI 0.000, 0.379). When considering only good-quality studies (24% of the Pb studies), only the relationship with hemoglobin levels remained (0.875; 95% CI: 0.546, 0.986).

**Conclusion:**

We found evidence of a negative relationship between lead concentration and hemoglobin and mean corpuscular hemoglobin and of a positive relationship with red cell distribution width in children. We also identified a need to conduct more studies in European countries. Future studies should use standardized practices and make efforts to increase study quality, namely by conducting comprehensive longitudinal studies. Our findings support the need to take further actions to limit heavy metal exposure during childhood.

## Introduction

Children are a high-risk group to heavy metals toxicity, due to increased susceptibility of their underdeveloped organ systems (1, 2). Children can be more exposed to heavy metals by spending more time outdoors compared to adults, frequent chewing of non-food items (1, 3), and higher consumption of food per kilogram of body weight (3). The main routes of exposure are the consumption of contaminated food and water, inhalation of contaminated air, and dermal absorption (1). Heavy metal toxicity in children has been associated with intellectual disability, neurocognitive, behavioral, and respiratory disorders, cancer, and cardiovascular diseases (1).

Human biomonitoring encompasses the measurement of the population's exposure to environmental pollutants and the assessment of their effects on health. The European Human Biomonitoring Initiative (HBM4EU) is a joint effort of 30 countries, the European Environment Agency, and the European Commission, co-funded under Horizon 2020. It aims to provide evidence of human exposure to chemicals and their links to health outcomes, supporting policy-making. This systematic review focuses on the five heavy metals prioritized by the HBM4EU initiative: lead (Pb), mercury (Hg), cadmium (Cd), arsenic (As), and chromium VI [Cr(VI)] (4). The prioritization process was based on the substances' hazardous properties, exposure characteristics (including environmental, consumer, and occupational exposure pathways), societal concerns, and policy needs (5).

The toxicity pathways of these heavy metals are similar, including inactivation of enzymes, generation of reactive oxygen species, weakening of antioxidant defense, and oxidative stress (6). Regarding damages to the hematopoietic system, heavy metals may induce toxicity directly to bone marrow precursors, inhibiting enzymes involved in cell division and maturation, impairment of red blood cell (RBC) transport, and immune-mediated cell destruction (7). Evidence from animal studies demonstrated that high concentrations of Pb, Hg, Cd, As, and Cr(VI) are associated with changes in some RBC parameters (8–12).

Globally, children (of both sexes) aged 0–9 years and female adolescents aged 15–19 years show the highest prevalence of anemia (13). The prevalence and years lived with disability attributed to anemia in children and adolescents are higher in countries with lower socio-demographic index (14). These countries, in turn, also show higher exposure to heavy metals (15).

To our knowledge, there are no systematic reviews of observational studies evaluating the relationship between these known pollutants and the hematopoietic system among children. Thus, this systematic review aims to summarize the available evidence of observational studies exploring the association between exposure (internal dose) to the HBM4EU prioritized heavy metals and RBC parameters in children.

## Methods

We performed a systematic review with a quantitative synthesis using vote counting based on the direction of the relationship (16). We followed the Preferred Reporting Items for Systematic Reviews and Meta-Analyses (PRISMA) guidelines (17). The protocol is provided in the Supplementary material.

### Eligibility criteria

Inclusion criteria of original peer-reviewed studies were defined as follows: 1) community-based observational studies (cohort, case-control with hematological conditions, or cross-sectional studies); 2) participants aged 18 years or younger; 3) heavy metals [Pb, Hg, Cd, As, and/or Cr(VI)] internal dose assessment; 4) assessment of RBC parameters [RBC count, hematocrit, hemoglobin, mean corpuscular volume (MCV), mean corpuscular hemoglobin (MCH), mean corpuscular hemoglobin concentration (MCHC), and red cell distribution width (RDW)]; and 5) full papers published since 1 January 2010. This timeframe was applied to focus on the most up-to-date evidence available, with more standardized methods, to increase comparability between studies.

Exclusion criteria were: 1) studies not written in English or Portuguese; 2) studies including patients with pre-specified health conditions not related to the heavy metal exposure (namely cancer, renal diseases, inherited metabolic, or blood disorders); and 3) studies including participants taking pharmacological substances during the investigation.

### Search strategy

We conducted a systematic review of the literature using PubMed, Scopus, and Web of Science electronic databases to identify studies regarding the relationship between Pb, Hg, Cd, As, and Cr(VI) and RBC parameters in children, published between 1 January 2010 and 12 April 2022.

The search was developed using a combination of key terms (MeSH, text word, and equivalents) related to the HBM4EU prioritized heavy metals and the RBC parameters considered relevant by the research team (Supplementary Table 1). Boolean operators (OR and AND), parentheses, quotation marks, and asterisks were used accordingly. Quotation marks were used to search for exact terms or expressions; parentheses were used to indicate a group of search terms or combine two or more groups of search terms enabling all possible combinations of sentences; asterisks (^*^) were used to search all words derived from the precedent inflected part. Publication date filters were applied. The full syntaxes used are presented in Supplementary Table 1. The searches were conducted in the online databases and the results were exported to a Microsoft Office Excel^®^ document (Microsoft Corporation, Washington, USA).

Additionally, we manually checked the lists of references of all included papers to identify additional literature.

### Selection process

After excluding duplicate records, all titles and abstracts of papers were screened independently by two authors (CC and RM) according to the inclusion/exclusion criteria. The screening process was performed using Microsoft Office Excel^®^. Full-text copies were retrieved and underwent an independent full-text review by the same two authors. Disagreements were resolved by consensus and, whenever consensus was not reached, by a third author (AV).

### Data collection process

Data from each paper were extracted independently by two authors (CC and RM), using a Microsoft Office Excel^®^ document, including the following information: title, author(s), year of publication, study design, study years, country, population, sex of participants, age (mean/median and range), type of biological sample, heavy metal/s, metal unit, metal concentration (mean/median and range), analytical method, source of exposure, RBC parameter/s, sample size (in the analysis), main findings, and adjustment variables/procedures. The two data extraction forms were compared, and disagreements were resolved by consensus.

Corresponding authors of each included paper were contacted to clarify and confirm the accuracy of the extracted data (response rate: 21%).

### Study quality assessment

Study quality was assessed using the National Institutes of Health's Study Quality Assessment (NIH-QA) Tools for Observational Cohort and Cross-Sectional Studies (18). This tool guides the author to focus on key concepts to a study's internal validity and has 14 items: 1) research question; 2) study population; 3) participation rate; 4) groups recruited from the same population and uniform eligibility criteria; 5) sample size justification; 6) temporal precedence of exposure; 7) sufficient timeframe to see an effect; 8) different levels of the exposure; 9) exposure measures clearly defined, valid, reliable, and implemented consistently across all study participants; 10) exposure assessed more than once over time; 11) outcome measures clearly defined, valid, reliable, and implemented consistently across all study participants; 12) blinding of outcome assessors; 13) follow-up rate; 14) adjustment of statistical analyses. Each item was rated as “yes,” “no,” “cannot determine”, “not reported”, or “not applicable”. Considering the risk of potential for selection bias, information bias, measurement bias, or confounding, author's classified each study as having “good”, “fair”, or “poor” quality. High risk of bias translated to a rating of poor quality and low risk of bias translated to a rating of good quality (18).

The quality of all studies was independently assessed by two authors (CC and RM). Disagreements were discussed and resolved by consensus.

### Synthesis methods

Results are organized by heavy metal and RBC parameters. Because of variation in study design, statistical analysis and outcome measures across studies, we synthesized the evidence using a vote counting approach based solely on the direction of effect (i.e., regardless of statistical significance or testing), as recommended by the Cochrane Foundation (16). A summary of each study's characteristics organized by heavy metal and RBC parameters is presented as Supplementary Tables.

When available data allowed, the direction of the relationship between heavy metal concentration and RBC parameter levels was defined for each study as “positive relationship”, “negative relationship”, or “conflicting results/no direction”. The categorization was done by assessing mean/median differences, correlation coefficients, odds ratios for having higher concentrations of heavy metals or RBC parameters, and/or linear regression coefficients. A “positive relationship” was reported when the heavy metal concentration was associated with higher levels of the RBC parameter (e.g., individuals with higher concentrations of a specific heavy metal presented a higher mean of an RBC parameter compared to individuals with lower concentrations, regardless of statistical significance). In contrast, a “negative relationship” was reported when the heavy metal concentration was associated with lower levels of the RBC parameter. “Conflicting results/no direction” was noted when studies reported relationships with opposite directions between subgroups (e.g., male/female, exposed/control group) or different biological samples (e.g., blood/erythrocyte), or when differences between means or correlation coefficients equal zero.

After categorizing each study, we compared the number of studies reporting negative relationships with the number of studies indicating positive relationships or conflicting results/no direction for each heavy metal and RBC parameter. An estimate of the proportion (*p*) of negative relationships was calculated as *p* = *u*/*n*, where *u* = number of negative relationships, and *n* = number of studies, along with a 95% confidence interval (CI), using Jeffrey's interval method. When the proportion is 0.5 (or the CI includes 0.5) the number of studies reporting negative and positive relationships is the same, meaning that there is no evidence of a specific relationship. A proportion higher than 0.5 means that there is evidence of a negative relationship between the two variables analyzed. Sensitivity analyses were conducted, in which we estimated the proportion of studies reporting negative relationships excluding the studies with conflicting results and excluding studies rated as having fair or poor quality. We presented the results from each study using effect direction plots.

## Results

### Study selection and description

**Figure 1:**
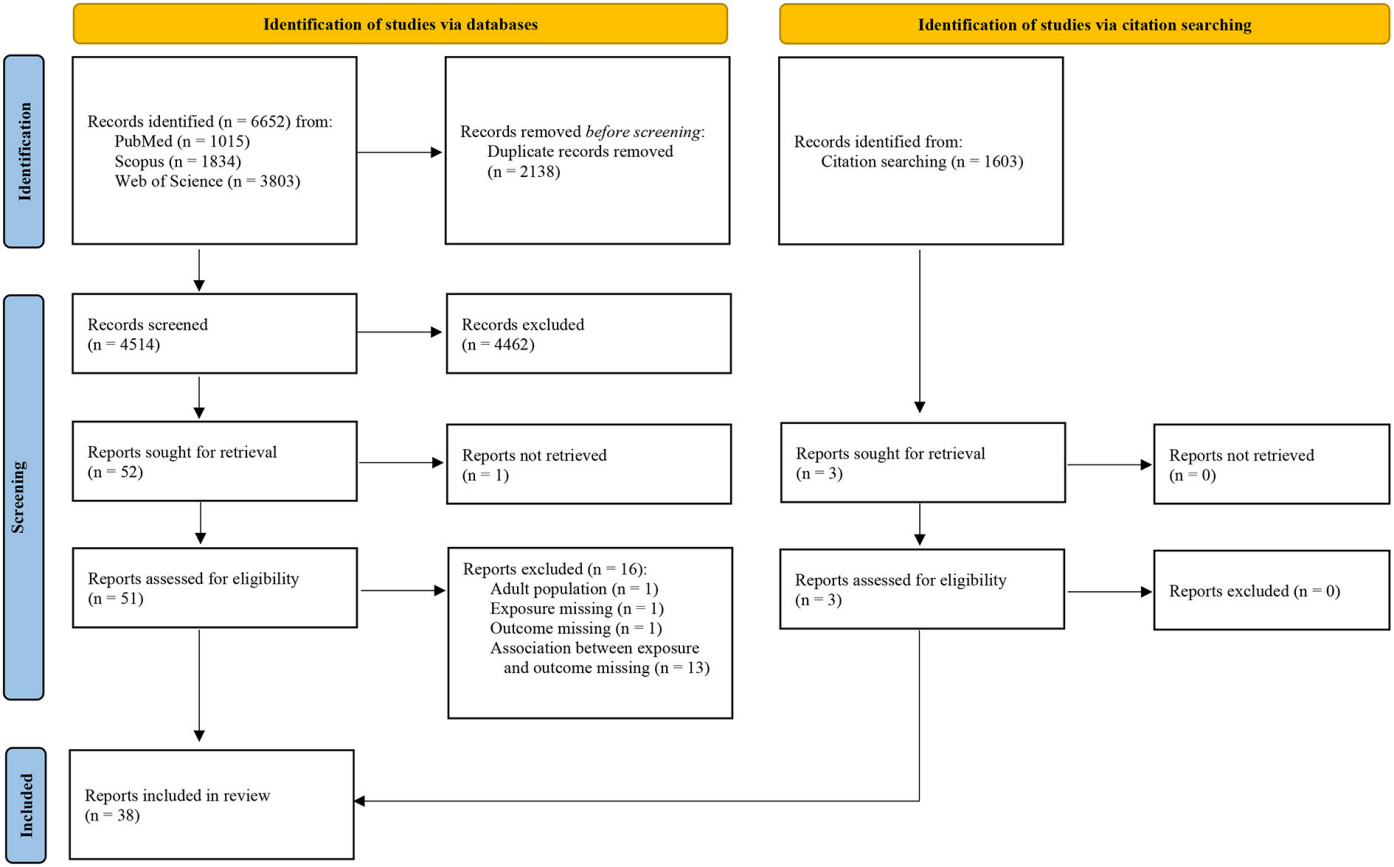
Study selection flow chart according to PRISMA 2020 guidelines (17).

Through our search, we identified 6,652 records. After excluding duplicates, we screened the title and abstract of 4,514 records and reviewed 51 full texts, of which, 35 papers met our eligibility criteria. By searching the citations of included studies (*n* = 1,662), we identified three additional papers meeting the inclusion criteria. Overall, we included 38 papers in this systematic review. The selection process and reasons for exclusion after full-text review are presented in Figure 1.

Of the 38 included papers, 33 assessed Pb concentrations (19–51), four Hg concentrations (52–55), two Cd concentrations (28, 37), and two As concentrations (28, 56). We did not identify any study exploring the association between Cr(VI) and RBC parameters in children published since 2010 in the selected electronic databases and fitting with the inclusion criteria.

### Study quality assessment

**Figure 2:**
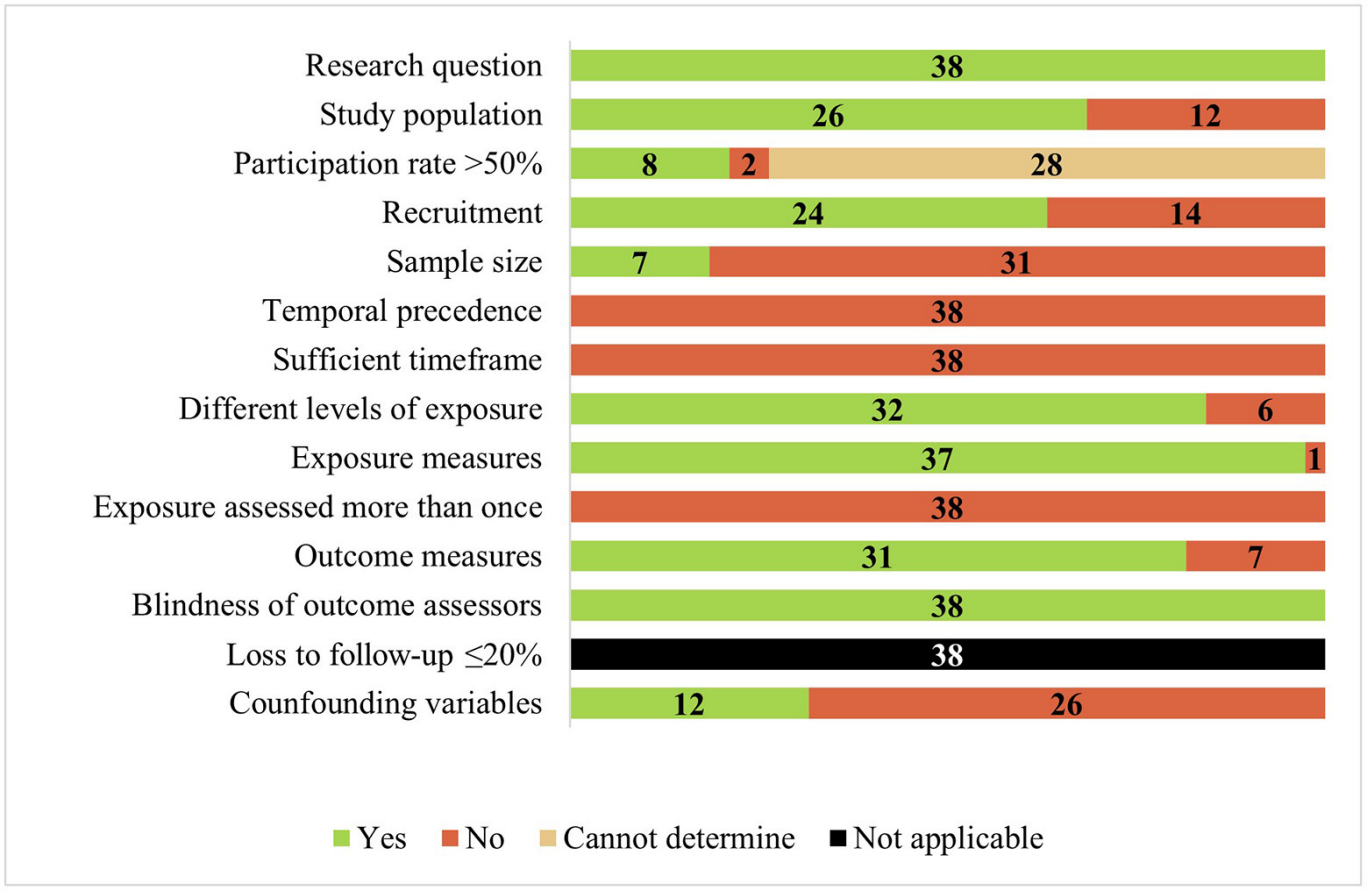
Quality assessment using national institutes of health's tool for observational cohort and cross-sectional studies (*n* = 38).

From the total, 11 studies were appraised as having good quality (23, 24, 30, 32, 35, 38, 41, 47, 52–54), nine as fair quality (26, 27, 31, 33, 42, 45, 46, 51, 55), and 18 as poor quality (19–22, 25, 28, 29, 34, 36, 37, 39, 40, 43, 44, 48–50, 56). Since nearly all included studies had a cross-sectional design, the most frequent methodological limitations were the lack of temporal precedence, sufficient timeframe between exposure and outcome, and exposure being assessed only once. Another frequent limitation was the lack of sample size calculations and statistical power. The studies' quality assessment by item is presented in Figure 2. Supplementary Table 2 shows the quality assessment by study.

### Lead

Overall, 33 papers published between 2010 and 2022 assessed the relationship between Pb concentration and RBC parameters in children (19–51). More than half (*n* = 21) were performed in Asia (22, 23, 25–27, 29, 31–36, 40, 43–47, 49–51), five in South America (20, 21, 28, 39, 41), four in Africa (19, 24, 37, 48), two in Europe (30, 42), and one in North America (38). Regarding study design, 30 studies are cross-sectional (19–23, 25–33, 35, 37–51), two are cross-sectional nested case-control (24, 34), and one is a cohort study (36). Regarding Pb exposure, 16 studies defined the source as environmental exposure (19, 20, 22, 25, 27, 30, 31, 33, 34, 40, 41, 43, 44, 48, 49, 51), with five related to e-waste recycling and disposal (27, 33, 40, 44, 51). Studies included a total of 36,205 participants (number of participants per study ranged between 43 and 17,486), aged between 0 and 18 years (mean/median age ranged between 1 and 12 years, among the studies reporting this data). Regarding biological samples, 26 studies reported blood Pb concentrations (19–21, 23–27, 29–32, 35, 38, 40–51), four reported both blood and erythrocyte Pb concentrations (22, 33, 36, 37), two studies reported capillary Pb concentrations (34, 39), and one study reported Pb concentration in hair (28). Pb concentrations were determined using graphite furnace atomic absorption spectrometry in 18 studies (20–22, 24, 26, 30–33, 36, 37, 41, 42, 44, 46–49), anodic stripping voltammetry in seven (27, 34, 35, 39, 40, 43, 51), inductively coupled plasma mass spectrometry in four (25, 28, 29, 38), atomic absorption spectrometry (type not specified) in two (19, 23), flame atomic absorption spectrometry in one study (45), and one study did not specify (50).

Of the 33 studies, seven explored the relationship between Pb concentration and RBC count (Supplementary Table 3) (20–22, 24, 29, 31, 40), eight studies with hematocrit (Supplementary Table 4) (20–22, 24, 29, 33, 38, 40), all 33 studies with hemoglobin (Supplementary Table 5) (19–51), nine studies with MCV (Supplementary Table 6) (20–22, 24, 26, 29, 38, 40, 42), eight with MCH (Supplementary Table 7) (20–22, 24, 26, 29, 31, 40), seven studies with MCHC (Supplementary Table 8) (20–22, 24, 26, 29, 40), and five studies with RDW (Supplementary Table 9) (20–22, 24, 40).

Vote counting based on the direction of the relationship between Pb concentration and RBC parameters by study and the proportion of studies reporting negative relationships between the two variables are presented in Table 1. We identified evidence of a relationship between Pb concentration and hemoglobin, MCH, and RDW. When only considering studies rated as having good quality, evidence regarding the relationship between Pb concentration and hemoglobin remained apparent.

**Table 1:** Vote counting based on the direction of the relationship between lead concentration and red blood cell parameters.

**Reference**	**Study**	**Sample size**	**Study quality**	**RBC count** ** (*n* = 7)**	**HCT** ** (*n* = 8)**	**HB** ** (*n* = 33)**	**MCV** ** (*n* = 9)**	**MCH** ** (*n* = 8)**	**MCHC** ** (*n* = 7)**	**RDW** ** (*n* = 5)**
(23)	Guo et al. 2021	17486	Good			▾				
(24)	Hegazy et al. 2010	60	Good	▴	▾	▾	▾	▾	▾	▴
(30)	Kutllovci-Zogaj et al. 2014	250	Good			▾				
(32)	Liu et al. 2012	140	Good			▾				
(35)	Mitra et al. 2012	559	Good			▾				
(38)	Ngueta, 2016	3799	Good		▴	▴	▾			
(41)	Rondó et al. 2011	384	Good			▾				
(47)	Zolaly et al. 2012	235	Good			▾				
(51)	Irawati et al. 2022	128	Fair			▴				
(26)	Keramati et al. 2013	223	Fair			▾	▴	▾	▾	
(27)	Khan et al. 2010	246	Fair			▾				
(31)	Li et al. 2018	743	Fair	▾		▾		▾		
(33)	Liu et al. 2015	855	Fair		▾	▾				
(42)	Ruiz-Tudela et al. 2021	1427	Fair			■	▴			
(45)	Wang et al. 2012	4429	Fair			▾				
(46)	Ye et al. 2015	1047	Fair			▾				
(20)	Alvarez-Ortega et al. 2017	118	Poor	▴	▾	▾	▾	▾	▾	▴
(21)	Alvarez-Ortega et al. 2019	554	Poor	▴	▴	▴	▴	▾	▾	▴
(50)	Banga et al. 2021	81	Poor			▴				
(22)	Dai et al. 2017	332	Poor	■	■	■	■	■	■	▴
(25)	Hoang et al. 2021	403	Poor			▾				
(28)	Kordas et al. 2010	222	Poor			▴				
(29)	Kuang et al. 2020	395	Poor	▴	▾	▾	▾	▾	▴	
(34)	Mitchell et al. 2012	67	Poor			■				
(48)	Moawad et al. 2016	300	Poor			▾				
(36)	Mohan et al. 2014	226	Poor			-				
(37)	Nassef et al. 2014	90	Poor			▾				
(39)	Queirolo et al. 2010	222	Poor			▾				
(19)	Rasoul et al. 2012	180	Poor			▾				
(40)	Rawat et al. 2021	43	Poor	▾	▾	▾	▾	▾	▾	▴
(43)	Reddy et al. 2011	195	Poor			▾				
(49)	Shah et al. 2010	340	Poor			▾				
(44)	Wang et al. 2021	426	Poor			▾				
**Proportion of negative relationships (95%CI)**										
All studies				0.286 (0.065, 0.648)	0.625 (0.295, 0.881)	**0.750 (0.583, 0.874)**	0.556 (0.254, 0.827)	**0.875 (0.546, 0.986)**	0.714 (0.352, 0.935)	**0.000 (0.000, 0.379)**
Excluding conflicting results				0.333 (0.077, 0.714)	0.714 (0.352, 0.935)	**0.828 (0.663, 0.931)**	0.625 (0.295, 0.881)	**1.000 (0.708, 1.000)**	0.833 (0.442, 0.981)	**0.000 (0.000, 0.379)**
Only good quality studies				0.000 (0.000, 0.853)	0.500 (0.061, 0.939)	**0.875 (0.546, 0.986)**	1.000 (0.333, 1.000)	1.000 (0.147, 1.000)	1.000 (0.147, 1.000)	0.000 (0.000, 0.853)

RBC, red blood cell; HCT, hematocrit; HB, hemoglobin; MCV, mean corpuscular volume; MCH, mean corpuscular hemoglobin; MCHC, mean corpuscular hemoglobin concentration; RDW, red cell distribution width; CI, confidence interval.

▴, positive relationship; ▾, negative relationship; ■, conflicting results/no direction; -, no information available.

Bolds represent evidence of a relationship.

### Mercury

We identified four cross-sectional studies assessing the relationship between Hg concentration and RBC parameters, published between 2017 and 2021 (52–55). Three studies were performed in South America (52–54) and one in Asia (55). Studies included a total of 1 971 participants, with more than half (*n* = 1 096) from one single study (55). Participants' age ranged between 0 and 18 years (mean/median age ranged between 1 and 14 years). Participants from one study lived near industrial activities (53); from another study, participants lived near resource extractive activities (54). The other two studies did not define the source of exposure. In three studies, Hg was assessed in hair (52–54), and in one study Hg was assessed in blood (55). Three studies determined Hg concentration using atomic absorption spectrometry (type not specified) (53–55), while the other study used cold vapor atomic absorption spectrometry (52).

One study assessed the relationship between Hg concentration and RBC count (Supplementary Table 10) (53), two with hematocrit (Supplementary Table 11) (53, 55), three with hemoglobin (Supplementary Table 12) (52–54), and one with MCHC (Supplementary Table 13) (53).

Vote counting based on the direction of the relationship between Hg concentration and RBC parameters by study and the proportion of studies reporting negative relationships between the two variables are presented in Table 2. Considering the proportion of studies reporting negative relationships, we did not identify evidence of a relationship with any RBC parameter.

**Table 2:** Vote counting based on the direction of the relationship between mercury concentration and red blood cell parameters.

**Reference**	**Study**	**Sample size**	**Study quality**	**RBC count** ** (*n* = 1)**	**HCT** ** (*n* = 2)**	**HB** ** (*n* = 3)**	**MCHC** ** (*n* = 1)**
(52)	Kempton et al. 2021	16	Good			▾	
(53)	Manjarres-Suarez and Olivero-Verbel, 2020	194	Good	▾	▾	▾	▾
(54)	Weinhouse et al. 2017	83	Good			▾	
(55)	Cho, 2021	1096	Fair		▴		
**Proportion of negative effects (95%CI)**						
All studies				1.000 (0.147, 1.000)	0.500 (0.061, 0.939)	1.00 (0.464, 1.000)	1.000 (0.147, 1.000)
Only good quality studies				1.000 (0.147, 1.000)	1.000 (0.147, 1.000)	1.00 (0.464, 1.000)	1.000 (0.147, 1.000)

RBC, red blood cell; HCT, hematocrit; HB, hemoglobin; MCHC, mean corpuscular hemoglobin concentration; CI, confidence interval.

▴, positive relationship; ▾, negative relationship.

### Cadmium

We identified two cross-sectional studies assessing the relationship between Cd -concentration and hemoglobin (Supplementary Table 14) (28, 37). Nassef et al. assessed 90 school children from Egypt with iron deficiency anemia aged between 6 and 12 years (37). Graphite furnace atomic absorption spectrometry was used to determine Cd in blood (37). Kordas et al. (28) assessed 222 preschool children from Uruguay aged between 0 and 3 years and assessed Cd concentration in hair using inductively coupled plasma mass spectrometry.

Considering the proportion of studies reporting a negative relationship between Cd concentration and hemoglobin, we did not find evidence of a relationship between the two variables (Table 3).

**Table 3:** Vote counting based on the direction of effect between cadmium concentration and red blood cell parameters.

**Reference**	**Study**	**Sample size**	**Study quality**	**HB (*n* = 2)**
(28)	Kordas et al. 2010	222	Poor	▴
(37)	Nassef et al. 2014	90	Poor	▾
**Proportion of negative effects (95%CI)**				0.500 (0.061, 0.939)

HB, hemoglobin; CI, confidence interval.

▴, positive relationship; ▾, negative relationship.

### Arsenic

Two cross-sectional studies assessed the relationship between As concentration and hemoglobin (Supplementary Table 15) (28, 56). The study performed by Kordas et al. (28) is described in the Cd section. Arsenic concentrations were assessed in hair using inductively coupled plasma mass spectrometry (28). López-Rodiguez et al. (56) assessed 40 children from Mexico aged between 6 and 12 years. Arsenic concentrations were assessed in blood using scanning electron microscopy/energy-dispersive X-ray spectrometry (56).

Similarly to Cd, we did not find evidence of a relationship between As concentration and hemoglobin (Table 4).

**Table 4:** Vote counting based on the direction of effect between arsenic concentration and red blood cell parameters.

**Reference**	**Study**	**Sample size**	**Study quality**	**HB** ** (*n* = 2)**
(28)	Kordas et al. 2010	222	Poor	▴
(56)	López-Rodríguez et al. 2017	40	Poor	▾
**Proportion of negative effects (95%CI)**				0.500 (0.061, 0.939)

HB, hemoglobin; CI, confidence interval.

▴, positive relationship; ▾, negative relationship.

## Discussion

In this systematic review, we summarize the available evidence assessing the relationship of exposure (internal dose) to heavy metals and RBC parameters in children. Understanding these relationships is essential to direct effective efforts aiming to decrease the burden associated with hematological conditions—namely anemia—in childhood. Assessment of RBC parameters is commonly available and allows clinicians to identify biochemical alterations before signs or symptoms are evident. When alterations are present, clinicians can discard heavy metal exposure as an influencing factor, especially in areas where there is an environmental concern. Also, if these parameters are defined as biomarkers of effect of heavy metal exposure, they would allow a more precise assessment of the effect on vulnerable populations, along with evaluating the impact of mitigation measures. In this systematic review, Pb is the most studied substance out of the five heavy metals prioritized by the HBM4EU initiative. The included studies had many methodological limitations, with only one-third having been rated as having good quality based on the NIH-QA Tools for Cohort and Cross-Sectional Studies. We found evidence of a relationship between Pb concentration and hemoglobin, MCV, and RDW, with only the first remaining when only considering studies rated as having good quality. Currently, the evidence between Hg, Cd, and As concentrations and RBC parameters is very scarce, which justifies the uncertainty of the estimated proportions of studies reporting negative relationships.

We did not identify any study regarding Cr(VI) published since 2010 in the selected databases, fitting the inclusion criteria. A possible explanation for this lack of studies is linked to the fact that Cr(VI) exposure is mainly occupational, with studies focusing on workers from chromium-related industries (57, 58). The RBC are a primary target for the exposure to Cr(VI) considering that this Cr-specie readily crosses the RBC membrane, whilst Cr(III) does not (59). Chromium exposure in chrome plating workers led to altered values of RBC count, Hb, and MCH (60), while in the general population hematological changes have been reported after ingestion of lethal or sublethal doses of Cr(VI) compounds (61, 62). However, because children may be exposed to Cr(VI)-polluted food, drinking water (61, 63), toys, or soils at uncontrolled hazardous waste sites (61), there is a need for studies assessing effect biomarkers of exposed children, including hematological ones.

More than half of the studies regarding Pb exposure were conducted in Asian countries. Lead toxicity is a public health concern in Asian countries (such as China and India) mainly due to the historical and current industrial pollution (64, 65), with children living near lead-contaminated industrial parks, public parks, kindergarten playgrounds, and commercial areas being at higher risk of exposure (65). Other sources of exposure are leaded paint, herbal products used in traditional medicines, jewelry, and native or traditional foods (65). Although heavy metal exposure is still a problem in Europe (65), we only identified two studies focusing on the investigation of the relationship between Pb concentration and RBC parameters in children.

The negative relationship between Pb concentration and hemoglobin found in the included observational studies is in line with the mechanistic evidence that Pb interferes with heme synthesis (66). Pb inhibits δ-aminolevulinic acid dehydratase enzyme, an enzyme that catalyzes the formation of porphobilinogen, a precursor of heme synthesis (66, 67). Pb also interferes with heme synthesis by inhibiting the enzyme ferrochelatase and, consequently, reducing the incorporation of iron into heme (66).

Lead has also been linked to other RBC parameters through other pathways, even though we did not find evidence of this relationship in children considering the included observational studies. These pathways include the pro-oxidative effect of δ-aminolevulinic acid that accumulates with the inhibition of the δ-aminolevulinic acid dehydratase enzyme and can be rapidly oxidized to generate free radicals (66). These free radicals, in turn, can cause oxidative damage to DNA fragments and membrane lipid peroxidation to RBC (66). Additionally, Pb can also promote hemolysis by inhibiting the two phosphoribosyl transferases in RBC (68).

The included studies were very heterogeneous with different aims, sample sizes, age ranges, sources of exposure, the biological sample analyzed, laboratory methods used, heavy metals concentrations, and data analysis methods, making comparisons between studies difficult. For this reason, other quantitative methods for data synthesis (e.g., meta-analysis) were not possible (16).

Most of the included studies have a cross-sectional design, not allowing to confirm the temporal precedence between the exposure and the outcome. Many studies also did not report or calculate the sample size or the statistical power of the sample, limiting the ability to extrapolate the results reported and assess the real meaning of the *p*-values reported. By using vote counting based on the direction of the relationship, we limited the impact that underpowered studies may have on the summarized results (16). However, our analysis provides no information on the magnitude of the relationships and does not account for differences in the relative sizes of the studies (16).

To ensure higher quality of the assessment, we included duplicate assessments at every stage, as recommended by PRISMA guidelines (17). However, additional studies could have been identified if additional databases were searched and papers in other languages were included. Yet, we mitigated this limitation by screening the references list of the included studies, by which we identified three additional studies. Since we only included published studies, publication bias cannot be ruled out. We only included papers published after 2010, to focus on the most up-to-date evidence available, which may already reflect (to a degree) the policies and regulations in place to mitigate environmental contamination by heavy metals such as efforts to reduce Hg (Minamata treaty) (3), the ban on leaded gasoline, the elimination of Pb solder from canned foods, and the ban on leaded paint (69, 70).

To date, several studies have explored the association between RBC parameters and heavy metals identified as HBM4EU priority substances in children, mainly regarding Pb exposure. However, studies were very heterogenous, most of them had a cross-sectional design, and only 11/38 studies were rated as having good quality. We found evidence of a negative relationship between Pb concentration and hemoglobin and MCH and a positive relationship with RDW. Only the relationship between Pb concentration and hemoglobin remained apparent when considering higher-quality studies. Efforts to standardize laboratory methods and biological samples used should be made to increase comparability between studies in future reviews. There is a need to conduct more studies in European countries, more comprehensive longitudinal studies (increasing study quality), as well as studies exploring the cumulative effect of different heavy metals. Our findings add to the body of evidence necessary to support the need to further limit environmental contamination by heavy metals through policies and regulations.

## Data availability statement

The original contributions presented in the study are included in the article/Supplementary material, further inquiries can be directed to the corresponding author.

## Author contributions

CC, RM, OS, MB, HT, and AV conceptualized the manuscript. CC, RM, and AV wrote the review protocol and performed the literature search. CC and RM extracted and interpreted the data and wrote the first draft of the manuscript. All authors reviewed and made valuable contributions to the manuscript.

## Funding

This work was developed under the HBM4EU initiative, funded by the European Union's Horizon 2020 Research and Innovation Programme under grant agreement no. 733032. The writing of the manuscript was also supported by funds from Fundação para a Ciência e a Tecnologia to ISAMB (ref. UIDB/04295/2020 and UIDP/04295/2020).

## Conflict of interest

The authors declare that the research was conducted in the absence of any commercial or financial relationships that could be construed as a potential conflict of interest.

## Publisher's note

All claims expressed in this article are solely those of the authors and do not necessarily represent those of their affiliated organizations, or those of the publisher, the editors and the reviewers. Any product that may be evaluated in this article, or claim that may be made by its manufacturer, is not guaranteed or endorsed by the publisher.
