# Supplementary material for: Exposure to heavy metals and red blood cell parameters in children: A systematic review of observational studies
Source: Front Pediatr. 2022 Oct 6;10:921239. doi: 10.3389/fped.2022.921239 (PMC9583003; doi:10.3389/fped.2022.921239)
Supplement: Supplementary file 1 [file Data_Sheet_1.docx]

Supplementary Material

Supplementary Table 1. Key terms and syntaxes used by electronic database

|  | | **Exposure (heavy metals)** | **Outcome (RBC parameters)** |
| --- | --- | --- | --- |
| **Keywords** | | Lead [MeSH term]  Lead metal  Lead chemical  Lead toxicity  Lead exposure  Cadmium [MeSH term]  Cadmium  Mercury [MeSH term]  Mercury  Arsenic [MeSH term]  Arsenic  Chromium hexavalent ion [MeSH term]  Chromium hexavalent ion  Cr(VI)  Chromium(VI)  Hexavalent chromium | Erythrocyte Count [MeSH term]  Erythrocyte*  Red Blood Cell*  Hematocrit [MeSH term]  Hematocrit*  Haematocrit*  Packed Red Cell Volume*  Hemoglobins [MeSH term]  Hemoglobin*  Haemoglobin*  Hemoglobulin*  Haemoglobulin*  Mean Corpuscular Volume*  Mean Cell Volume*  Red Cell Distribution Width |
| **Syntaxes** | **PubMed** | ((((((((((((((((Lead[MeSH Terms]) OR (Cadmium[MeSH Terms])) OR (Mercury[MeSH Terms])) OR (Arsenic[MeSH Terms])) OR (Chromium hexavalent ion[MeSH Terms])) OR (Lead metal[Title/Abstract])) OR (Lead chemical[Title/Abstract])) OR (Lead toxicity[Title/Abstract])) OR (Lead exposure[Title/Abstract])) OR (Cadmium[Title/Abstract])) OR (Mercury[Title/Abstract])) OR (Arsenic[Title/Abstract])) OR (Chromium hexavalent ion[Title/Abstract])) OR ("Cr(VI)"[Title/Abstract])) OR ("Chromium(VI)"[Title/Abstract])) OR (Hexavalent chromium[Title/Abstract])) AND (((((((((((((((Erythrocyte Count[MeSH Terms]) OR (Hematocrit[MeSH Terms])) OR (Hemoglobins[MeSH Terms])) OR (Erythrocyte*[Title/Abstract])) OR (Red Blood Cell*[Title/Abstract])) OR (Hematocrit*[Title/Abstract])) OR (Haematocrit*[Title/Abstract])) OR (Packed Red Cell Volume*[Title/Abstract])) OR (Hemoglobin*[Title/Abstract])) OR (Haemoglobin*[Title/Abstract])) OR (Hemoglobulin*[Title/Abstract])) OR (Haemoglobulin*[Title/Abstract])) OR (Mean Corpuscular Volume*[Title/Abstract])) OR (Mean Cell Volume*[Title/Abstract])) OR (Red Cell Distribution Width[Title/Abstract])) | |
|  | **Scopus** | ( TITLE-ABS-KEY ( "lead metal" ) OR TITLE-ABS-KEY ( "lead chemical" ) OR TITLE-ABS-KEY ( "lead toxicity" ) OR TITLE-ABS-KEY ( "lead exposure" ) OR TITLE-ABS-KEY ( "cadmium" ) OR TITLE-ABS-KEY ( "mercury" ) OR TITLE-ABS-KEY ( "arsenic" ) OR TITLE-ABS-KEY ( "Chromium hexavalent ion" ) OR TITLE-ABS-KEY ( "Cr(VI)" ) OR TITLE-ABS-KEY ( "Chromium(VI)" ) OR TITLE-ABS-KEY ( "Hexavalent chromium" ) ) AND ( TITLE-ABS-KEY ( erythrocyte* ) OR TITLE-ABS-KEY ( red AND blood AND cell* ) OR TITLE-ABS-KEY ( hematocrit* ) OR TITLE-ABS-KEY ( haematocrit* ) OR TITLE-ABS-KEY ( packed AND red AND cell AND volume* ) OR TITLE-ABS-KEY ( hemoglobin* ) OR TITLE-ABS-KEY ( haemoglobin* ) OR TITLE-ABS-KEY ( hemoglobulin* ) OR TITLE-ABS-KEY ( haemoglobulin* ) OR TITLE-ABS-KEY ( mean AND corpuscular AND volume* ) OR TITLE-ABS-KEY ( mean AND cell AND volume* ) OR TITLE-ABS-KEY ( red AND cell AND distribution AND width ) ) | |
|  | **Web of Science** | (((((((((((TS=(Lead metal)) OR TS=(Lead chemical)) OR TS=(Lead toxicity)) OR TS=(Lead exposure)) OR TS=(Cadmium)) OR TS=(Mercury)) OR TS=(Arsenic)) OR TS=(Chromium hexavalent ion)) OR TS=(Cr(VI))) OR TS=(Chromium(VI))) OR TS=(Hexavalent chromium)) AND (TS=(Erythrocyte*) OR TS=(Red Blood Cell*) OR TS=(Hematocrit*) OR TS=(Haematocrit*) OR TS=(Packed Red Cell Volume*) OR TS=(Hemoglobin*) OR TS=(Haemoglobin*) OR TS=(Hemoglobulin*) OR TS=(Haemoglobulin*) OR TS=(Mean Corpuscular Volume*) OR TS=(Mean Cell Volume*) OR TS=(Red Cell Distribution Width)) | |

Supplementary Table 2. Study quality assessment using the National Institutes of Health’s Study Quality Assessment Tools for Observational Cohort and Cross-Sectional Studies

| **Study** | **Research question** | **Study population** | **Participation rate >50%** | **Recruitment** | **Sample size** | **Temporal precedence** | **Sufficient timeframe** | **Different levels of exposure** | **Exposure measures** | **Exposure assessed >1x** | **Outcome measures** | **Blindness of assessors** | **Loss to follow-up ≤20%** | **Confounding variables** | **Criteria**  **fulfillment** | **Overall** |
| --- | --- | --- | --- | --- | --- | --- | --- | --- | --- | --- | --- | --- | --- | --- | --- | --- |
| Alvarez-Ortega, et al. 2017 |  |  |  |  |  |  |  |  |  |  |  |  |  |  | 46% | Poor |
| Alvarez-Ortega, et al. 2019 |  |  |  |  |  |  |  |  |  |  |  |  |  |  | 46% | Poor |
| Banga, et al. 2021 |  |  |  |  |  |  |  |  |  |  |  |  |  |  | 38% | Poor |
| Cho, 2021 |  |  |  |  |  |  |  |  |  |  |  |  |  |  | 54% | Fair |
| Dai, et al. 2017 |  |  |  |  |  |  |  |  |  |  |  |  |  |  | 38% | Poor |
| Guo, et al. 2021 |  |  |  |  |  |  |  |  |  |  |  |  |  |  | 62% | Good |
| Hegazy, et al. 2010 |  |  |  |  |  |  |  |  |  |  |  |  |  |  | 69% | Good |
| Hoang, et al. 2021 |  |  |  |  |  |  |  |  |  |  |  |  |  |  | 38% | Poor |
| Irawati, et al. 2022 |  |  |  |  |  |  |  |  |  |  |  |  |  |  | 54% | Fair |
| Kempton, et al. 2021 |  |  |  |  |  |  |  |  |  |  |  |  |  |  | 62% | Good |
| Keramati, et al. 2013 |  |  |  |  |  |  |  |  |  |  |  |  |  |  | 54% | Fair |
| Khan, et al. 2010 |  |  |  |  |  |  |  |  |  |  |  |  |  |  | 54% | Fair |
| Kordas, et al. 2010 |  |  |  |  |  |  |  |  |  |  |  |  |  |  | 38% | Poor |
| Kuang, et al. 2020 |  |  |  |  |  |  |  |  |  |  |  |  |  |  | 38% | Poor |
| Kutllovci-Zogaj, et al. 2014 |  |  |  |  |  |  |  |  |  |  |  |  |  |  | 62% | Good |
| Li, et al. 2018 |  |  |  |  |  |  |  |  |  |  |  |  |  |  | 54% | Fair |
| Liu, et al. 2015 |  |  |  |  |  |  |  |  |  |  |  |  |  |  | 54% | Fair |
| Liu, et al. 2012 |  |  |  |  |  |  |  |  |  |  |  |  |  |  | 62% | Good |
| López-Rodríguez, et al. 2017 |  |  |  |  |  |  |  |  |  |  |  |  |  |  | 46% | Poor |
| Manjarres-Suarez & Olivero-Verbel, 2020 |  |  |  |  |  |  |  |  |  |  |  |  |  |  | 62% | Good |
| Mitchell, et al. 2012 |  |  |  |  |  |  |  |  |  |  |  |  |  |  | 38% | Poor |
| Mitra, et al. 2012 |  |  |  |  |  |  |  |  |  |  |  |  |  |  | 62% | Good |
| Moawad, et al. 2016 |  |  |  |  |  |  |  |  |  |  |  |  |  |  | 38% | Poor |
| Mohan, et al. 2014 |  |  |  |  |  |  |  |  |  |  |  |  |  |  | 38% | Poor |
| Nassef, et al. 2014 |  |  |  |  |  |  |  |  |  |  |  |  |  |  | 46% | Poor |
| Ngueta, 2016 |  |  |  |  |  |  |  |  |  |  |  |  |  |  | 69% | Good |
| Queirolo, et al. 2010 |  |  |  |  |  |  |  |  |  |  |  |  |  |  | 54% | Poor |
| Rasoul, et al. 2012 |  |  |  |  |  |  |  |  |  |  |  |  |  |  | 38% | Poor |
| Rawat, et al. 2021 |  |  |  |  |  |  |  |  |  |  |  |  |  |  | 46% | Poor |
| Rondó, et al. 2011 |  |  |  |  |  |  |  |  |  |  |  |  |  |  | 62% | Good |
| Ruiz-Tudela, et al. 2021 |  |  |  |  |  |  |  |  |  |  |  |  |  |  | 62% | Fair |
| Reddy, et al. 2011 |  |  |  |  |  |  |  |  |  |  |  |  |  |  | 46% | Poor |
| Shah, et al. 2010 |  |  |  |  |  |  |  |  |  |  |  |  |  |  | 38% | Poor |
| Wang, et al. 2021 |  |  |  |  |  |  |  |  |  |  |  |  |  |  | 46% | Poor |
| Wang, et al. 2012 |  |  |  |  |  |  |  |  |  |  |  |  |  |  | 54% | Fair |
| Weinhouse, et al. 2017 |  |  |  |  |  |  |  |  |  |  |  |  |  |  | 62% | Good |
| Ye, et al. 2015 |  |  |  |  |  |  |  |  |  |  |  |  |  |  | 54% | Fair |
| Zolaly, et al. 2012 |  |  |  |  |  |  |  |  |  |  |  |  |  |  | 62% | Good |

Yes No Cannot determine Not applicable

Supplementary Table 3. Summary of studies assessing the association between lead concentration and red blood cell count

| **Study** | **Design** | **Data collection timeframe** | **Country** | **Population** | **Sex included** | **Age, mean/ [median]** | **Age, range/ [IQR]** | **Source of exposure** | **Biological sample** | **Determination method** | **n (analysis)** | **Metal unit** | **Metal levels, mean (SD/SE)/ [median]** | **Metal levels, range/[IQR]** | **Association** | **Adjusted** | **Direction** |
| --- | --- | --- | --- | --- | --- | --- | --- | --- | --- | --- | --- | --- | --- | --- | --- | --- | --- |
| Alvarez-Ortega et al., 2017 | Cross-sectional | 2014 | Colombia | Children living near melting activities | All | - | 5-16 | Environmental (living near melting activities) | Whole blood | Graphite furnace atomic absorption spectrometry | 118 | μg/dL | 1.7 (0.3) | 0.05-34.05 | Spearman correlation: rho=0.012; p=0.899 | No | ▲ |
| Alvarez-Ortega et al., 2019 | Cross-sectional | 2015 | Colombia | Children | All | - | 5-16 | - | Venous blood | Graphite furnace atomic absorption spectrometry | 554 | μg/dL | 3.5 (0.2) | 0.1-50.1 | Spearman correlation: rho=0.037; p=0.385 | No | ▲ |
| Dai et al., 2017 | Cross-sectional | 2015 | China | Kindergarten children | All | 4 | 2-6 | Environmental (e-waste-exposed area) | Whole blood | Graphite furnace atomic absorption spectrometry | 332 | μg/dL | Exposed: [6.5]  Reference: [4.5] | Exposed: [5.1-8.1]  Reference: [3.4-5.6] | Pearson correlation in exposed: r=0.030; p=0.592  Pearson correlation in reference: r=-0.019; p=0.816 | No | ■ |
|  |  |  |  |  |  |  |  |  | Erythrocyte | Graphite furnace atomic absorption spectrometry | 332 | μg/dL | Exposed: [17.0]  Reference: [11.9] | Exposed: [13.0-21.4]  Reference: [9.2-15.1] | Pearson correlation in exposed: r=-0.046, p= 0.402  Pearson correlation in reference: r=-0.069; p=0.396 | No |  |
| Hegazy et al., 2010 | Cross-sectional nested case-control | - | Egypt | Children | All | 6 | 2-14 | - | Venous blood | Graphite furnace atomic absorption spectrometry | 60 | μg/dL | - | 7-20 | Correlation: r=0.118; p=0.37 | No | ▲ |
| Kuang et al., 2020 | Cross-sectional | 2012 | China | Students from public primary schools in an industrial city | Male | - | 7-11 | - | Venous blood | Inductively coupled plasma mass spectrometry | 219 | μg/L | [27.1] | 20.9-38.4 | Linear regression for RBC (1012/L): β=0.018; p=0.156; R2=0.009 | Yes - Picky eaters, passive smoking in family and outdoor sports duration | ▲ |
|  |  |  |  |  | Female |  |  |  |  |  | 176 | μg/L | [24.5] | 17.5-35.5 | Linear regression for RBC (1012/L): β=0.005; p=0.701; R2=0.001 |  |  |
| Li et al., 2018 | Cross-sectional | 2012-2017 | China | Children from the Blood Lead Intervention Program | All | - | 5-8 | Environmental | Whole blood | Graphite furnace atomic absorption spectrometry | 743 | μg/L | 82.4 | P5-P95: 28.8-218.2 | Logistic regression for RBC (boys: <4.0 ×1012/L; girls: <3.5 ×1012/L) with increasing BLL by 1µg/L: OR=1.01 (1.00, 1.01) \| with BLL≥100 vs. <100: OR=2.72 (0.99, 7.53) | Yes - Age, gender, BMI, environmental lead exposure level, and serum contents of iron, zinc, and calcium | ▼ |
| Rawat et al., 2021 | Cross-sectional | - | India | Elementary school children near an informal battery recycling manufacturing unit | All | 7 | 4-12 | Environmental (battery recycling manufacturing unit) | Venous blood | Anodic stripping voltammetry | 43 | μg/dL | 19.93 (9.22) | 9.1-37.4 | Pearson correlation: r=-0.603; p<0.001 | No | ▼ |

Supplementary Table 4. Summary of studies assessing the association between lead concentration and hematocrit

| **Study** | **Design** | **Data collection timeframe** | **Country** | **Population** | **Sex included** | **Age, mean/ [median]** | **Age, range/ [IQR]** | **Source of exposure** | **Biological sample** | **Determination method** | **n (analysis)** | **Metal unit** | **Metal levels, mean (SD/SE)/ [median]** | **Metal levels, range/[IQR]** | **Association** | **Adjusted** | **Direction** |
| --- | --- | --- | --- | --- | --- | --- | --- | --- | --- | --- | --- | --- | --- | --- | --- | --- | --- |
| Alvarez-Ortega et al., 2017 | Cross-sectional | 2014 | Colombia | Children living near melting activities | All | - | 5-16 | Environmental (living near melting activities) | Whole blood | Graphite furnace atomic absorption spectrometry | 118 | μg/dL | 1.7 (0.3) | 0.05-34.05 | Spearman correlation: rho=-0.078; p=0.403 | No | ▼ |
| Alvarez-Ortega et al., 2019 | Cross-sectional | 2015 | Colombia | Children | All | - | 5-16 | - | Venous blood | Graphite furnace atomic absorption spectrometry | 554 | μg/dL | 3.5 (0.2) | 0.1-50.1 | Spearman correlation: rho=0.119; p=0.006 | No | ▲ |
| Dai et al., 2017 | Cross-sectional | 2015 | China | Kindergarten children | All | 4 | 2-6 | Environmental (e-waste-exposed area) | Whole blood | Graphite furnace atomic absorption spectrometry | 332 | μg/dL | Exposed: [6.5]  Reference: [4.5] | Exposed: [5.1-8.1]  Reference: [3.4-5.6] | Pearson correlation in exposed: r=-0.168; p=0.002  Pearson correlation in reference: r=0.030; p=0.718  Multiple linear regression for BLL in exposed group: β=-0.001 (-0.002, 0.001) | Yes (regression) - Gender and age | ■ |
|  |  |  |  |  |  |  |  |  | Erythrocyte |  |  |  | Exposed: [17.0]  Reference: [11.9] | Exposed: [13.0-21.4]  Reference: [9.2-15.1] | Pearson correlation in exposed: r=-0.295; p=0.000  Pearson correlation in reference: r=-0.135; p=0.098  Multiple linear regression for Erythrocyte Pb level in exposed group: β=-0.001 (-0.001, -0.001) |  |  |
| Hegazy et al., 2010 | Cross-sectional nested case-control | - | Egypt | Children | All | 6 | 2-14 | - | Venous blood | Graphite furnace atomic absorption spectrometry | 60 | μg/dL | - | 7-20 | Correlation: r=-0.484; p<0.01 | No | ▼ |
| Kuang et al., 2020 | Cross-sectional | 2012 | China | Students from public primary schools in an industrial city | Male | - | 7-11 | - | Venous blood | Inductively coupled plasma mass spectrometry | 219 | μg/L | [27.1] | 20.9-38.4 | Linear regression for HCT (%): β=-0.041; p=0.019; R2=0.025 | Yes - Picky eaters, passive smoking in family and outdoor sports duration | ▼ |
|  |  |  |  |  | Female |  |  |  |  |  | 176 | μg/L | [24.5] | 17.5-35.5 | Linear regression for HCT (%): β=-0.023; p=0.330; R2=0.005 |  |  |
| Liu et al., 2015 | Cross-sectional | 2006-2011 | China | Children from areas with industry related to e-waste processing | All | - | 3-7 | Environmental (e-waste processing) | Blood | Graphite furnace atomic absorption spectrometry | 855  (Exposed: 435, Controls: 420) | μg/dL | Exposed: [8.19]  Controls: [6.72]  p<0.01 | 2.20-37.78 | Spearman correlation: rho=-0.05; p>0.05 | No | ▼ |
|  |  |  |  |  |  |  |  |  | Erythrocyte |  |  |  | Exposed: [21.95]  Controls: [17.32]  p<0.01 | 5.98-101.01 | Spearman correlation: rho=-0.19; p<0.001 |  |  |
| Ngueta, 2016 | Cross-sectional | 2007-2011 | Canada | Participants aged between 6-18 years from the Canadian Health and Measures Survey | All | 12 | 6-18 | - | Blood | Inductively coupled plasma mass spectrometry | 3799 | μg/dL | 0.79 | - | Mean (95%CI) BLL: HCT (%) ≥34 vs. <34 = 0.89 (0.85-0.93) vs. 0.77 (0.65-0.92); p=0.1203 | Yes - Age (continuous), gender, ethnicity (non-Hispanic Whites, non-Hispanic Blacks, others), body mass index, age of residence, and blood calcium concentration and income level | ▲ |
| Rawat et al., 2021 | Cross-sectional | - | India | Elementary school children near an informal battery recycling manufacturing unit | All | 7 | 4-12 | Environmental (battery recycling manufacturing unit) | Venous blood | Anodic stripping voltammetry | 43 | μg/dL | 19.93 (9.22) | 9.1-37.4 | Pearson correlation: r=-0.624; p<0.001 | No | ▼ |

Supplementary Table 5. Summary of studies assessing the association between lead concentration and hemoglobin

| **Study** | **Design** | **Data collection timeframe** | **Country** | **Population** | **Sex included** | **Age, mean/ [median]** | **Age, range/ [IQR]** | **Source of exposure** | **Biological sample** | **Determination method** | **n (analysis)** | **Metal unit** | **Metal levels, mean (SD/SE)/ [median]** | **Metal levels, range/[IQR]** | **Association** | **Adjusted** | **Direction** |
| --- | --- | --- | --- | --- | --- | --- | --- | --- | --- | --- | --- | --- | --- | --- | --- | --- | --- |
| Alvarez-Ortega et al., 2017 | Cross-sectional | 2014 | Colombia | Children living near melting activities | All | - | 5-16 | Environmental (living near melting activities) | Whole blood | Graphite furnace atomic absorption spectrometry | 118 | μg/dL | 1.7 (0.3) | 0.05-34.05 | Spearman correlation: rho=-0.145; p=0.117 | No | ▼ |
| Alvarez-Ortega et al., 2019 | Cross-sectional | 2015 | Colombia | Children | All | - | 5-16 | - | Venous blood | Graphite furnace atomic absorption spectrometry | 554 | μg/dL | 3.5 (0.2) | 0.1-50.1 | Spearman correlation: rho=0.033; p=0.436 | No | ▲ |
| Banga et al., 2021 | Cross-sectional | 2019-2020 | India | Children showing clinical signs of anemia | All | - | 1-5 | - | Venous blood | - | 81 | μg/dL | - | - | %Hb<11: BLL<5 vs. BLL≥5 = 66.7% vs. 51.3%  Linear regression for Hb (g/dL): β=0.007376; p=0.0853 | No | ▲ |
| Dai et al., 2017 | Cross-sectional | 2015 | China | Kindergarten children | All | 4 | 2-6 | Environmental (e-waste-exposed area) | Whole blood | Graphite furnace atomic absorption spectrometry | 332 | μg/dL | Exposed: [6.5]  Reference: [4.5] | Exposed: [5.1-8.1]  Reference: [3.4-5.6] | Pearson correlation in exposed: r=-0.158; p=0.004  Pearson correlation in reference: r= 0.031; p=0.706  Multiple linear regression for BLL in exposed group: β=-0.628 (-0.953, -0.304) | Yes (regression) - Gender and age | ▼ |
|  |  |  |  |  |  |  |  |  | Erythrocyte |  |  |  | Exposed: [17.0]  Reference: [11.9] | Exposed: [13.0-21.4]  Reference: [9.2-15.1] | Pearson correlation in exposed: r=-0.279; p=0.000  Pearson correlation in reference: r=-0.121; p=0.136  Multiple linear regression for Erythrocyte Pb level in exposed group: β=-0.333 (-0.443, -0.222) |  |  |
| Guo et al., 2021 | Cross-sectional | 2014-2017 | China | Children | All | 2 | 0-5 | - | Blood | Atomic absorption spectrometry | 17486 | μg/L | 31.50 (16.61) | - | Mean (SD) Hb (g/L): BLL <16.05 = 119.65 (10.30) \| BLL 16.05-24.37 = 120.14 (10.44) \| BLL 24.38-33.34 = 120.79 (10.89) \| BLL 33.35-45.00 = 120.69 (10.89) \| BLL >45.00 = 120.76 (11.22); p<0.001  Differences in Hb (g/L): BLL 16.05-24.37 vs. <16.05 = -0.050 (-0.505, 0.404) \| BLL 24.38-33.34 vs. <16.05 = -0.023 (-0.479, 0.432) \| BLL 33.35-45.00 vs. <16.05 = - 0.489 (-0.943, -0.035) \| BLL> 45.00 vs. <16.05 = -1.245 (-1.708, -0.783)  Correlation: r=-0.047; p<0.05 | Yes (correlation) - Age and sex | ▼ |
| Hegazy et al., 2010 | Cross-sectional nested case-control | - | Egypt | Children | All | 6 | 2-14 | - | Venous blood | Graphite furnace atomic absorption spectrometry | 60 | μg/dL | - | 7-20 | %Hb <11g/dL: BLL <10 vs. ≥10 = 27.3% vs. 63.2%; p<0.01  %Hb 10-10.9 g/dL: BLL <10 vs. ≥10 = 4.5 vs. 28.9; p<0.05 \| %Hb 8-9.9 g/dL = 18.3 vs. 13.2; p>0.05 \| %Hb <8g/dL = 4.5 vs. 21.1; p<0.05 \| p=0.01  Correlation: r=-0.461; p<0.01 | No | ▼ |
| Hoang et al., 2021 | Cross-sectional | 2016 | Vietnam | Children living in a 10-km radius around zinc-lead mining | All | 8 | 3-14 | Environmental (villages contaminated by hydrogeological and geochemical lead–zinc mines) | Blood | Inductively coupled plasma mass spectrometry | 403  (Location 1: 195, Location 2: 208) | μg/dL | Location 1: 15.42 (6.45)  Location 2: 13.47 (11.48)  p < 0.001 | Location 1: 4.97-37.99  Location 2: 0.1-61.49 | Pearson correlation in location 1: r=-0.079 (-1.174, 0.016); p=0.10  Pearson correlation in location 2: r=-0.036 (-0.190, 0.117); p=0.64 | No | ▼ |
| Irawati et al., 2022 | Cross-sectional | 2019 | Indonesia | Children living in four hamlets where used lead-acid batteries used to be recycled | All | 3 | 1-5 | Environmental (living near former used lead batteries recycling) | Blood | Anodic Stripping Voltammetry | 128 | μg/dL | 17.03 (11.78) | 4.0-65.0 | %Hb<11g/dL: BLL>10 vs. <10 = 33.7% vs. 33.3%  OR for Hb<11g/dL with BLL >10 = 1.017 (95%CI: 0.458, 2.258) | No | ▲ |
| Keramati et al., 2013 | Cross-sectional | 2010 | Iran | Children | All | 5 | 1-10 | - | Blood | Graphite furnace atomic absorption spectrometry | 223 | μg/dL | 57 (22.7) | 10.9-212 | Correlation: r=-0.09; p=0.186 | No | ▼ |
| Khan et al., 2010 | Cross-sectional | - | Pakistan | Children of smelters and battery recycle workers living close to the industries | All | 4 | 1-6 | Environmental (children of smelters and battery recycle workers living close to the industries) | Venous blood | Anodic stripping voltammetry | 246 | μg/dL | Exposed: [8.1]  Controls: [6.7]  p<0.01 | Exposed: 1.0-20.9  Controls: 1.4-13.3 | Spearman correlation: rho=-0.34; p=0.001 | No | ▼ |
| Kordas et al., 2010 | Cross-sectional | 2007 | Uruguay | Preschool children | All | 2 | 0-3 | - | Hair | Inductively coupled plasma mass spectrometry | 222 | μg/g | 17.64 (17.43) | 0.44-161.70 | Multivariate regression for log-transformed hair Pb levels: β=0.28 (0.15); p<0.1 | Yes - Age, sex, blood lead, maternal education, household possessions, and maternal hair metal level | ▲ |
| Kuang et al., 2020 | Cross-sectional | 2012 | China | Students from public primary schools in an industrial city | Male | - | 7-11 | - | Venous blood | Inductively coupled plasma mass spectrometry | 219 | μg/L | [27.1] | 20.9-38.4 | Linear regression for Hb (g/L): β=-0.119; p=0.017; R2=0.026 | Yes - Picky eaters, passive smoking in family and outdoor sports duration | ▼ |
|  |  |  |  |  | Female |  |  |  |  |  | 176 |  | [24.5] | 17.5-35.5 | Linear regression for Hb (g/L): β=-0.082; p=0.280; R2=0.007 |  |  |
| Kutllovci-Zogaj et al., 2014 | Cross-sectional | - | Kosovo | Kindergarten and primary school children | All | Primary school: 8  Primary school control group: 9  Kindergarten: 5 | 5-12 | Environmental (pollution) | Blood | Graphite furnace atomic absorption spectrometry | 250  (Primary school: 166,  Primary school controls: 53, Kindergarten: 31) | μg/dL | Primary school: 2.4 (1.9)  Primary school control group: 2.3 (0.7)  Kindergarten: 3.8 (1.3) | Primary school: 0.5-16.3  Primary school controls: 1.2-5.2  Kindergarten: 2.2-7.7 | Spearman correlation: rho=-0305; p<0.0001 | No | ▼ |
| Li et al., 2018 | Cross-sectional | 2012-2017 | China | Children from the Blood Lead Intervention Program | All | - | 5-8 | Environmental | Whole blood | Graphite furnace atomic absorption spectrometry | 743 | μg/L | 82.4 | P5-P95: 28.8-218.2 | Logistic regression for Hb<115g/L with increasing BLL by 1µg/L: OR=1.00 (1.00, 1.01) \| with BLL≥100 vs. <100: OR=2.51 (1.38, 4.57) | Yes - Age, gender, BMI, environmental lead exposure level, and serum contents of iron, zinc, and calcium | ▼ |
| Liu et al., 2015 | Cross-sectional | 2006-2011 | China | Children from areas with industry related to e-waste processing | All | - | 3-7 | Environmental (e-waste processing) | Blood | Graphite furnace atomic absorption spectrometry | 855  (Exposed: 435, Controls: 420) | μg/dL | Exposed: [8.19]  Controls: [6.72]  p<0.01 | 2.20-37.78 | Spearman correlation: rho=-0.05; p>0.05  Mean difference (95%CI) Hb (g/L): BLL Q1 = Ref \| Q2 = -0.63 (-2.35, 1.10) \| Q3 = 0.78 (-0.95, 2.51) \| Q4 = 1.45 (-0.28, 3.18) | Yes - Age, sex, residence area, and SES | ▼ |
|  |  |  |  |  |  |  |  |  | Erythrocyte |  |  |  | Exposed: [21.95]  Controls: [17.32]  p<0.01 | 5.98-101.01 | Spearman correlation: rho=-0.16; p<0.001  Mean difference (95%CI) Hb (g/L): EPb Q1 = Ref \| Q2 = -0.02 (-1.89, 1.52) \| Q3=-3.01 (-4.71, 1.31) \| Q4=-3.97 (-5.68, -2.27)  Mean difference (95%CI) per doubling of Epb = -2.44 (-2.01, -2.86) |  |  |
| Liu et al., 2012 | Cross-sectional | - | China | Preschool children from the China Jintan Child Cohort Study | All | 3 | - | - | Blood | Graphite furnace atomic absorption spectrometry | 140 | μg/dL | [4.3] | 1.9-11.4 | Linear regression for Hb: β=-0.096 (-0.179, 0.012) | Yes - Age, gender, height, weight, and Fe deficiency | ▼ |
| Mitchell et al., 2012 | Cross-sectional nested case-control | 2009 | Thailand-Burma border | US-bound refugee children | All | - | 0-14 | Environmental | Capillary blood, with confirmation of BLL >10 by venous blood | Anodic stripping voltammetry | 67  (<2 years: 30, ≥2 years: 37) | μg/dL | - | Cases: ≥10  Controls: <3.3 | Children <2y: median (range) Hb (g/dL) BLL ≥10 vs. <3.3 = 9.7 (7.0-12.1) vs. 10.5 (9.3-12.7); p=0.054  Multivariate regression for BLL ≥10 with Hb<10g/dL = 26.6 (2.9, undefined); p=0.0024  Children ≥2y: median (range) Hb BLL≥10 vs. <3.3 = 11.8 (10.1-14.2) vs. 11.7 (6.0-16.5); p=0.49 | Yes (regression) - Exposure to motor vehicle batteries and traditional medicine | ■ |
| Mitra et al., 2012 | Cross-sectional | 2007-2009 | Bangladesh | Preschool and school children | All | - | <16 | - | Venous blood | Anodic stripping voltammetry | 559 | μg/dL | - | <10-69 | Pearson correlation: r=-0.102; p=0.016 | No | ▼ |
| Mohan et al., 2014 | Cohort | 2010-2012 | India | Pregnant women enrolled in a multi-country study on malnutrition, a birth cohort (called the Mal-ED) | All | 1 | 1 | - | Venous blood | Graphite furnace atomic absorption spectrometry | 226 | μg/dL | 10.3 (5.0) | 2.4-29.7 | Correlation between Hb and BLL at 15 months: p>0.05 | No | - |
| Nassef et al., 2014 | Cross-sectional | - | Egypt | School children with iron deficiency anemia | All | 10 | 6-12 | - | Blood | Graphite furnace atomic absorption spectrometry | 90 | nmol/L | Male: 1.23 (0.23)  Female: 1.44 (0.23) | - | Pearson correlation: r=-0.811; p<0.01 | No | ▼ |
| Ngueta, 2016 | Cross-sectional | 2007-2011 | Canada | Participants aged between 6-18 years from the Canadian Health and Measures Survey | All | 12 | 6-18 | - | Blood | Inductively coupled plasma mass spectrometry | 3799 | μg/dL | 0.79 | - | Mean (95%CI) BLL: Hb (g/L) ≥112 vs. <112 = 0.89 (0.85-0.93) vs. 0.69 (0.56-0.83); p=0.0085 | Yes - Age (continuous), gender, ethnicity (non-Hispanic Whites, non-Hispanic Blacks, others), body mass index, age of residence, and blood calcium concentration and income level | ▲ |
| Queirolo et al., 2010 | Cross-sectional | 2007 | Uruguay | Children 6 to 36 months | All | 2 | 0-4 | - | Capillary blood | Anodic stripping voltammetry | 222 | μg/dL | 9.0 (6.0) | 1-35.6 | Mean (SD) BLL: Hb ≥10.5g/dL vs. <10.5 = 7.9 (5.1) vs. 10.4 (6.8); p<0.01  Multiple linear regression for BLL by having Hb < 10.5g/dL: β= 2.40 (0.77, 4.03)  Logistic regression for BLL ≥10μg/dL by having Hb < 10.5g/dL: OR= 1.90 (1.08, 3.35) | Yes (regressions) - Age and fingers/toys | ▼ |
| Rasoul et al., 2012 | Cross-sectional | - | Egypt | Primary school children | All | - | - | Environmental (urban districts) | Blood | Atomic absorption spectrometry | 180 | μg/dL | 6.72 (4.3) | - | Mean (SD) Hb (g/dL): BLL<10 vs. ≥10 = 12.63 (1.02) vs. 12.11 (1.12); p=0.004  Pearson correlation: r=-0.196; p=0.03 | No | ▼ |
| Rawat et al., 2021 | Cross-sectional | - | India | Elementary school children near an informal battery recycling manufacturing unit | All | 7 | 4-12 | Environmental (battery recycling manufacturing unit) | Venous blood | Anodic stripping voltammetry | 43 | μg/dL | 19.93 (9.22) | 9.1-37.4 | Pearson correlation: r=-0.639; p<0.001 | No | ▼ |
| Reddy et al., 2011 | Cross-sectional | - | India | Apparently healthy school children | All | 12 | 9-14 | Environmental (urban, urban heavy traffic, and urban industrial) | Venous blood | Anodic stripping voltammetry | 195 | μg/dL | 11.8 (11.96) | 1.1-78.8 | Pearson correlation: r=-0.066; p>0.05 | No | ▼ |
| Rondó et al., 2011 | Cross-sectional | 2001-2003 | Brazil | Preschool and school children participating in a prospective cohort epidemiological study | All | 7 | 2-11 | Environmental (lead poisoning area) | Venous blood | Graphite furnace atomic absorption spectrometry | 384 | μg/dL | 6.99 (4.39) | - | Multivariate linear regression for BLL: β=-0.589 (-1.080, -0.099) | Yes - Distance from home to lead-manipulating industry and ferritin | ▼ |
| Ruiz-Tudela et al., 2021 | Cross-sectional | 2007-2009 | Spain | Healthy children | All | 8 | 1-16 | - | Venous blood | Graphite furnace atomic absorption spectrometry | 1427  (BLL<5: 1396, ≥5µg/dL: 31) | μg/dL | 1.98 (1.1) | P2.5-P97.5: 1-4 | Mean (SD) Hb (g/dL): BLL<5 vs. ≥5 = 13.3 (1.0) vs. 13.3 (1.1); p=0.77 | No | ■ |
| Wang et al., 2021 | Cross-sectional | 2014 | China | Kindergarten children | All | 5 | 3-6 | Environmental (e-waste disposal area) | Venous blood | Graphite furnace atomic absorption spectrometry | 426  (Exposed: 222, Controls: 204) | μg/dL | Exposed: [8.5]  Controls: [6.0]  p=0.00 | Exposed: [6.6-10.9]  Controls: [4.8-7.9] | Mean (SD) Hb (g/L) of exposed group: BLL <5.0 vs. 5.0-9.9 vs. ≥10.0 = 124.9 (8.2) vs. 122.6 (9.5) vs. 120.3 (7.3); p=0.03  Mean (SD) Hb (g/L) of control group: BLL <5.0 vs. 5.0-9.9 vs. ≥10.0 = 126.9 (8.5) vs. 125.8 (8.2) vs. 123.6 (8.3); p=0.14 | No | ▼ |
| Wang et al., 2012 | Cross-sectional | 2008-2011 | China | Healthy children | All | - | 0-7 | - | Fasting venous blood | Flame atomic absorption spectrometry | 4429 | μg/dL | 6.17 (2.292) | 1.0-26.0 | Pearson correlation: r=-0.020; p=0.185 | No | ▼ |
| Ye et al., 2015 | Cross-sectional | 2012-2013 | China | Children | All | - | 0-6 | - | Venous blood | Graphite furnace atomic absorption spectrometry | 1047 | μg/L | 33.72 (19.03) | 4.72-96.49 | Mean (SD) Hb (g/dL): BLL<50 vs. ≥50 = 12.87 (1.57) vs. 12.47 (1.48); p<0.01  Partial correlation: r=-0.040; p>0.05 | Yes (correlation) - Age, gender, and residents | ▼ |
| Zolaly et al., 2012 | Cross-sectional | 2010 | Saudi Arabia | Saudi school students (elementary, intermediate, and secondary) | All | - | 6-12 | - | Blood | Graphite furnace atomic absorption spectrometry | 235 | μg/dL | 4.94 (3.38) | - | Mean (SD) Hb (mg/dL): BLL<10 vs. ≥10 = 12.40 (1.17) vs. 9.33 (0.94); p=0.000 | No | ▼ |
|  |  |  |  |  | Male | - | 12-18 |  |  |  | 172 |  |  |  | Mean (SD) Hb (mg/dL): BLL <10 vs. ≥10 = 13.45 (0.90) vs. 10.55 (1.27); p=0.000 |  |  |
|  |  |  |  |  | Female |  |  |  |  |  | 150 |  |  |  | Mean (SD) Hb (mg/dL): BLL <10 vs. ≥10 = 12.31 (1.11) vs. 10.42 (1.26); p=0.000 |  |  |

Supplementary Table 6. Summary of studies assessing the association between lead concentration and mean corpuscular volume

| **Study** | **Design** | **Data collection timeframe** | **Country** | **Population** | **Sex included** | **Age, mean/ [median]** | **Age, range/ [IQR]** | **Source of exposure** | **Biological sample** | **Determination method** | **n (analysis)** | **Metal unit** | **Metal levels, mean (SD/SE)/ [median]** | **Metal levels, range/[IQR]** | **Association** | **Adjusted** | **Direction** |
| --- | --- | --- | --- | --- | --- | --- | --- | --- | --- | --- | --- | --- | --- | --- | --- | --- | --- |
| Alvarez-Ortega et al., 2017 | Cross-sectional | 2014 | Colombia | Children living near melting activities | All | - | 5-16 | Environmental (living near melting activities) | Whole blood | Graphite furnace atomic absorption spectrometry | 118 | μg/dL | 1.7 (0.3) | 0.05-34.05 | Spearman correlation: rho=-0.103; p=0.267 | No | ▼ |
| Alvarez-Ortega et al., 2019 | Cross-sectional | 2015 | Colombia | Children | All | - | 5-16 | - | Venous blood | Graphite furnace atomic absorption spectrometry | 554 | μg/dL | 3.5 (0.2) | 0.1-50.1 | Spearman correlation: rho=0.124; p=0.004 | No | ▲ |
| Dai et al., 2017 | Cross-sectional | 2015 | China | Kindergarten children | All | 4 | 2-6 | Environmental (e-waste-exposed area) | Whole blood | Graphite furnace atomic absorption spectrometry | 332 | μg/dL | Exposed: [6.5]  Reference: [4.5] | Exposed: [5.1-8.1]  Reference: [3.4-5.6] | Pearson correlation in exposed: r=-0.204; p=0.000  Pearson correlation in reference: r=0.041; p=0.613  Multiple linear regression for BLL in exposed group: β=-0.333 (-0.504, -0.162) | Yes (regression) - Gender and age | ■ |
|  |  |  |  |  |  |  |  |  | Erythrocyte |  |  |  | Exposed: [17.0]  Reference: [11.9] | Exposed: [13.0-21.4]  Reference: [9.2-15.1] | Pearson correlation in exposed: r=-0.237; p=0.000  Pearson correlation in reference: r=-0.024; p=0.772  Multiple linear regression for Erythrocyte Pb level in exposed group: β=-0.138 (-0.198, -0.079) |  |  |
| Hegazy et al., 2010 | Cross-sectional nested case-control | - | Egypt | Children | All | 6 | 2-14 | - | Venous blood | Graphite furnace atomic absorption spectrometry | 60 | μg/dL | - | 7-20 | Correlation: r=-0.267; p=0.03 | No | ▼ |
| Keramati et al., 2013 | Cross-sectional | 2010 | Iran | Children | All | 5 | 1-10 | - | Blood | Graphite furnace atomic absorption spectrometry | 223 | μg/dL | 57 (22.7) | 10.9-212 | Correlation: r= 0.051; p=0.457 | No | ▲ |
| Kuang et al., 2020 | Cross-sectional | 2012 | China | Students from public primary schools in an industrial city | All | - | 7-11 | - | Venous blood | Inductively coupled plasma mass spectrometry | 395 | μg/L | [26.1] |  | Linear regression for MCV (%): β=-0.041; p=0.000; R2=0.052 | Yes - Picky eaters, passive smoking in family and outdoor sports duration | ▼ |
| Ngueta, 2016 | Cross-sectional | 2007-2011 | Canada | Participants aged between 6-18 years from the Canadian Health and Measures Survey | All | 12 | 6-18 | - | Blood | Inductively coupled plasma mass spectrometry | 3799 | μg/dL | 0.79 | - | Mean (95%CI) BLL: MCV (fL) ≥73 vs. <73 = 0.88 (0.84-0.92) vs. 0.97 (0.83-1.14); p=0.2378 | Yes - Age (continuous), gender, ethnicity (non-Hispanic Whites, non-Hispanic Blacks, others), body mass index, age of residence, and blood calcium concentration and income level | ▼ |
| Rawat et al., 2021 | Cross-sectional | - | India | Elementary school children near an informal battery recycling manufacturing unit | All | 7 | 4-12 | Environmental (battery recycling manufacturing unit) | Venous blood | Anodic stripping voltammetry | 43 | μg/dL | 19.93 (9.22) | 9.1-37.4 | Pearson correlation: r=-0.668; p<0.001 | No | ▼ |
| Ruiz-Tudela et al., 2021 | Cross-sectional | 2007-2009 | Spain | Healthy children | All | 8 | 1-16 | - | Venous blood | Graphite furnace atomic absorption spectrometry | 1427  (BLL<5: 1396, ≥5µg/dL: 31) | μg/dL | 1.98 (1.1) | P2.5-P97.5: 1-4 | Mean (SD) MCV (fL): BLL<5 vs. ≥5 = 77.7 (3.9) vs. 78.9 (6.1); p=0.30 | No | ▲ |

Supplementary Table 7. Summary of studies assessing the association between lead concentration and mean corpuscular hemoglobin

| **Study** | **Design** | **Data collection timeframe** | **Country** | **Population** | **Sex included** | **Age, mean/ [median]** | **Age, range/ [IQR]** | **Source of exposure** | **Biological sample** | **Determination method** | **n (analysis)** | **Metal unit** | **Metal levels, mean (SD/SE)/ [median]** | **Metal levels, range/[IQR]** | **Association** | **Adjusted** | **Direction** |
| --- | --- | --- | --- | --- | --- | --- | --- | --- | --- | --- | --- | --- | --- | --- | --- | --- | --- |
| Alvarez-Ortega et al., 2017 | Cross-sectional | 2014 | Colombia | Children living near melting activities | All | - | 5-16 | Environmental (living near melting activities) | Whole blood | Graphite furnace atomic absorption spectrometry | 118 | μg/dL | 1.7 (0.3) | 0.05-34.05 | Spearman correlation: rho=-0.168; p=0.069 | No | ▼ |
| Alvarez-Ortega et al., 2019 | Cross-sectional | 2015 | Colombia | Children | All | - | 5-16 | - | Venous blood | Graphite furnace atomic absorption spectrometry | 554 | μg/dL | 3.5 (0.2) | 0.1-50.1 | Spearman correlation: rho=-0.023; p=0.591 | No | ▼ |
| Dai et al., 2017 | Cross-sectional | 2015 | China | Kindergarten children | All | 4 | 2-6 | Environmental (e-waste-exposed area) | Whole blood | Graphite furnace atomic absorption spectrometry | 332 | μg/dL | Exposed: [6.5]  Reference: [4.5] | Exposed: [5.1-8.1]  Reference: [3.4-5.6] | Pearson correlation in exposed: r=-0.178; p=0.001  Pearson correlation in reference: r=0.037; p=0.653  Multiple linear regression for BLL in exposed group: β=-0.140 (-0.206, -0.074) | Yes (regression) - Gender and age | ■ |
|  |  |  |  |  |  |  |  |  | Erythrocyte |  |  |  | Exposed: [17.0]  Reference: [11.9] | Exposed: [13.0-21.4]  Reference: [9.2-15.1] | Pearson correlation in exposed: r=-0.211; p=0.000  Pearson correlation in reference: r=-0.031; p=0.709  Multiple linear regression for Erythrocyte Pb level in exposed group: β=-0.057 (-0.080, -0.034) |  |  |
| Hegazy et al., 2010 | Cross-sectional nested case-control | - | Egypt | Children | All | 6 | 2-14 | - | Venous blood | Graphite furnace atomic absorption spectrometry | 60 | μg/dL | - | 7-20 | Correlation: r=-0.381; p<0.01 | No | ▼ |
| Keramati et al., 2013 | Cross-sectional | 2010 | Iran | Children | All | 5 | 1-10 | - | Blood | Graphite furnace atomic absorption spectrometry | 223 | μg/dL | 57 (22.7) | 10.9-212 | Correlation: r=-0.007; p=0.913 | No | ▼ |
| Kuang et al., 2020 | Cross-sectional | 2012 | China | Students from public primary schools in an industrial city | All | - | 7-11 | - | Venous blood | Inductively coupled plasma mass spectrometry | 395 | μg/L | [26.1] |  | Linear regression for MCH (pg): β=-0.012; p=0.004; R2=0.015 | Yes - Picky eaters, passive smoking in family and outdoor sports duration | ▼ |
| Li et al., 2018 | Cross-sectional | 2012-2017 | China | Children from the Blood Lead Intervention Program | All | - | 5-8 | Environmental | Whole blood | Graphite furnace atomic absorption spectrometry | 743 | μg/L | 82.4 | P5-P95: 28.8-218.2 | Logistic regression for MCH <27 pg with increasing BLL by 1µg/L: OR=1.01 (1.00, 1.01) | Yes - Age, gender, BMI, environmental lead exposure level, and serum contents of iron, zinc, and calcium | ▼ |
| Rawat et al., 2021 | Cross-sectional | - | India | Elementary school children near an informal battery recycling manufacturing unit | All | 7 | 4-12 | Environmental (battery recycling manufacturing unit) | Venous blood | Anodic stripping voltammetry | 43 | μg/dL | 19.93 (9.22) | 9.1-37.4 | Pearson correlation: r=-0.642; p<0.001 | No | ▼ |

Supplementary Table 8. Summary of studies assessing the association between lead concentration and mean corpuscular hemoglobin concentration

| **Study** | **Design** | **Data collection timeframe** | **Country** | **Population** | **Sex included** | **Age, mean/ [median]** | **Age, range/ [IQR]** | **Source of exposure** | **Biological sample** | **Determination method** | **n (analysis)** | **Metal unit** | **Metal levels, mean (SD/SE)/ [median]** | **Metal levels, range/[IQR]** | **Association** | **Adjusted** | **Direction** |
| --- | --- | --- | --- | --- | --- | --- | --- | --- | --- | --- | --- | --- | --- | --- | --- | --- | --- |
| Alvarez-Ortega et al., 2017 | Cross-sectional | 2014 | Colombia | Children living near melting activities | All | - | 5-16 | Environmental (living near melting activities) | Whole blood | Graphite furnace atomic absorption spectrometry | 118 | μg/dL | 1.7 (0.3) | 0.05-34.05 | Spearman correlation: rho=-0.004; p=0.962 | No | ▼ |
| Alvarez-Ortega et al., 2019 | Cross-sectional | 2015 | Colombia | Children | All | - | 5-16 | - | Venous blood | Graphite furnace atomic absorption spectrometry | 554 | μg/dL | 3.5 (0.2) | 0.1-50.1 | Spearman correlation: rho=-0.224; p<0.001 | No | ▼ |
| Dai et al., 2017 | Cross-sectional | 2015 | China | Kindergarten children | All | 4 | 2-6 | Environmental (e-waste-exposed area) | Whole blood | Graphite furnace atomic absorption spectrometry | 332 | μg/dL | Exposed: [6.5]  Reference: [4.5] | Exposed: [5.1-8.1]  Reference: [3.4-5.6] | Pearson correlation in exposed: r=-0.011; p=0.847  Pearson correlation in reference: r=0.008; p=0.924  Multiple linear regression for BLL in exposed group: β=-0.392 (-0.716, -0.069) | Yes (regression) - Gender and age | ■ |
|  |  |  |  |  |  |  |  |  | Erythrocyte |  |  |  | Exposed: [17.0]  Reference: [11.9] | Exposed: [13.0-21.4]  Reference: [9.2-15.1] | Pearson correlation in exposed: r=-0.027; p=0.629  Pearson correlation in reference: r=-0.036; p=0.656  Multiple linear regression for Erythrocyte Pb level in exposed group: β=-0.153 (-0.267, -0.039) |  |  |
| Hegazy et al., 2010 | Cross-sectional nested case-control | - | Egypt | Children | All | 6 | 2-14 | - | Venous blood | Graphite furnace atomic absorption spectrometry | 60 | μg/dL | - | 7-20 | Correlation: r=-0.155; p=0.23 | No | ▼ |
| Keramati et al., 2013 | Cross-sectional | 2010 | Iran | Children | All | 5 | 1-10 | - | Blood | Graphite furnace atomic absorption spectrometry | 223 | μg/dL | 57 (22.7) | 10.9-212 | Correlation: r=-0.096; p=0.156 | No | ▼ |
| Kuang et al., 2020 | Cross-sectional | 2012 | China | Students from public primary schools in an industrial city | All | - | 7-11 | - | Venous blood | Inductively coupled plasma mass spectrometry | 395 | μg/L | [26.1] |  | Linear regression for MCHC (g/L): β=0.255; p=0.575; R2=0.001 | Yes - Picky eaters, passive smoking in family and outdoor sports duration | ▲ |
| Rawat et al., 2021 | Cross-sectional | - | India | Elementary school children near an informal battery recycling manufacturing unit | All | 7 | 4-12 | Environmental (battery recycling manufacturing unit) | Venous blood | Anodic stripping voltammetry | 43 | μg/dL | 19.93 (9.22) | 9.1-37.4 | Pearson correlation: r=-0.597; p<0.001 | No | ▼ |

Supplementary Table 9. Summary of studies assessing the association between lead concentration and red cell distribution width

| **Study** | **Design** | **Data collection timeframe** | **Country** | **Population** | **Sex included** | **Age, mean/ [median]** | **Age, range/ [IQR]** | **Source of exposure** | **Biological sample** | **Determination method** | **n (analysis)** | **Metal unit** | **Metal levels, mean (SD/SE)/ [median]** | **Metal levels, range/[IQR]** | **Association** | **Adjusted** | **Direction** |
| --- | --- | --- | --- | --- | --- | --- | --- | --- | --- | --- | --- | --- | --- | --- | --- | --- | --- |
| Alvarez-Ortega et al., 2017 | Cross-sectional | 2014 | Colombia | Children living near melting activities | All | - | 5-16 | Environmental (living near melting activities) | Whole blood | Graphite furnace atomic absorption spectrometry | 118 | μg/dL | 1.7 (0.3) | 0.05-34.05 | Spearman correlation: rho=0.195; p=0.035 | No | ▲ |
| Alvarez-Ortega et al., 2019 | Cross-sectional | 2015 | Colombia | Children | All | - | 5-16 | - | Venous blood | Graphite furnace atomic absorption spectrometry | 554 | μg/dL | 3.5 (0.2) | 0.1-50.1 | Spearman correlation: rho=0.002; p=0.967 | No | ▲ |
| Dai et al., 2017 | Cross-sectional | 2015 | China | Kindergarten children | All | 4 | 2-6 | Environmental (e-waste-exposed area) | Whole blood | Graphite furnace atomic absorption spectrometry | 332 | μg/dL | Exposed: [6.5]  Reference: [4.5] | Exposed: [5.1-8.1]  Reference: [3.4-5.6] | Pearson correlation in exposed: r=0.132; p=0.016  Pearson correlation in reference: r=0.018; p=0.826  Multiple linear regression for BLL in exposed group: β=0.027 (-0.001, 0.056) | Yes (regression) - Gender and age | ▲ |
|  |  |  |  |  |  |  |  |  | Erythrocyte |  |  | μg/dL | Exposed: [17.0]  Reference: [11.9] | Exposed: [13.0-21.4]  Reference: [9.2-15.1] | Pearson correlation in exposed: r=0.162; p=0.003  Pearson correlation in reference: r=0.086; p=0.292  Multiple linear regression for Erythrocyte Pb level in exposed group: β=0.011 (0.001, 0.021) |  |  |
| Hegazy et al., 2010 | Cross-sectional nested case-control | - | Egypt | Children | All | 6 | 2-14 | - | Venous blood | Graphite furnace atomic absorption spectrometry | 60 | μg/dL | - | 7-20 | Correlation: r=0.458; p<0.01 | No | ▲ |
| Rawat et al., 2021 | Cross-sectional | - | India | Elementary school children near an informal battery recycling manufacturing unit | All | 7 | 4-12 | Environmental (battery recycling manufacturing unit) | Venous blood | Anodic stripping voltammetry | 43 | μg/dL | 19.93 (9.22) | 9.1-37.4 | Pearson correlation: r=0.555; p<0.001 | No | ▲ |

Supplementary Table 10. Summary of studies assessing the association between mercury concentration and red blood cell count

| **Study** | **Design** | **Data collection timeframe** | **Country** | **Population** | **Sex included** | **Age, mean/ [median]** | **Age, range/ [IQR]** | **Source of exposure** | **Biological sample** | **Determination method** | **n (analysis)** | **Metal unit** | **Metal levels, mean (SD/SE)/ [median]** | **Metal levels, range/[IQR]** | **Association** | **Adjusted** | **Direction** |
| --- | --- | --- | --- | --- | --- | --- | --- | --- | --- | --- | --- | --- | --- | --- | --- | --- | --- |
| Manjarres-Suarez & Olivero-Verbel, 2020 | Cross-sectional | - | Colombia | Adolescents | All | - | 11-18 | Environmental (industrial areas) | Hair | Atomic absorption spectrometry | 194 | μg/g | 1.43 (0.07) | 0.04-4.81 | Spearman correlation: rho=-0.009; p=0.897 | No | ▼ |

Supplementary Table 11. Summary of studies assessing the association between mercury concentration and hematocrit

| **Study** | **Design** | **Data collection timeframe** | **Country** | **Population** | **Sex included** | **Age, mean/ [median]** | **Age, range/ [IQR]** | **Source of exposure** | **Biological sample** | **Determination method** | **n (analysis)** | **Metal unit** | **Metal levels, mean (SD/SE)/ [median]** | **Metal levels, range/[IQR]** | **Association** | **Adjusted** | **Direction** |
| --- | --- | --- | --- | --- | --- | --- | --- | --- | --- | --- | --- | --- | --- | --- | --- | --- | --- |
| Cho, 2021 | Cross-sectional | 2010-2013 | Korea | Children and adolescents from KNHANES | All | 14 | 10-18 | - | Whole blood | Atomic absorption spectrometry | 1096 | µg/L | Normal weight: 2.09 (0.04)  Overweight: 2.43 (0.08)  p<0.05 | - | Geomeans (95% CI) HCT (%): Hg Q1 = 1.98 (1.74–2.22) \| Q2 = 2.14 (1.88–2.39) \| Q3 = 2.25 (2.01–2.49) \| Q4 = 2.24 (1.99–2.45); p<0.05 between Q3 and Q1 and between Q4 and Q1 | Yes - Age, sex, seafood consumption, household income, smoking, drinking, and blood hematocrit levels | ▲ |
| Manjarres-Suarez & Olivero-Verbel, 2020 | Cross-sectional | - | Colombia | Adolescents | All | - | 11-18 | Environmental (industrial areas) | Hair | Atomic absorption spectrometry | 194 | μg/g | 1.43 (0.07) | 0.04-4.81 | Spearman correlation: rho=-0.047; p=0.517 | No | ▼ |

Supplementary Table 12. Summary of studies assessing the association between mercury concentration and hemoglobin

| **Study** | **Design** | **Data collection timeframe** | **Country** | **Population** | **Sex included** | **Age, mean/ [median]** | **Age, range/ [IQR]** | **Source of exposure** | **Biological sample** | **Determination method** | **n (analysis)** | **Metal unit** | **Metal levels, mean (SD/SE)/ [median]** | **Metal levels, range/[IQR]** | **Association** | **Adjusted** | **Direction** |
| --- | --- | --- | --- | --- | --- | --- | --- | --- | --- | --- | --- | --- | --- | --- | --- | --- | --- |
| Kempton et al., 2021 | Cross-sectional | 2019 | Brazil | Mothers and children from Indigenous villages | All | 1 | 0-2 | - | Hair | Cold vapor atomic absorption spectrometry | 16 | μg/g | 3.88 (3.05) | <19.6 | Spearman correlation: r=-0.265; p=0.431 | No | ▼ |
| Manjarres-Suarez & Olivero-Verbel, 2020 | Cross-sectional | - | Colombia | Adolescents | All | - | 11-18 | Environmental (industrial areas) | Hair | Atomic absorption spectrometry | 194 | μg/g | 1.43 (0.07) | 0.04-4.81 | Spearman correlation: rho=-0.067; p=0.352 | No | ▼ |
| Weinhouse et al., 2017 | Cross-sectional | 2015 | Peru | Children from the Amarakaeri Reserve Cohort Study | All | - | <12 | Environmental (near resource extractive activities) | Hair | Atomic absorption spectrometry | 83 | μg/g | [1.18] | 0.06-9.70 | Linear regression for Hb: β=-0.14; p=0.04 | Yes - Age, sex, HAZ, WHZ, and vitamin B12 | ▼ |

Supplementary Table 13. Summary of studies assessing the association between mercury concentration and mean corpuscular hemoglobin concentration

| **Study** | **Design** | **Data collection timeframe** | **Country** | **Population** | **Sex included** | **Age, mean/ [median]** | **Age, range/ [IQR]** | **Source of exposure** | **Biological sample** | **Determination method** | **n (analysis)** | **Metal unit** | **Metal levels, mean (SD/SE)/ [median]** | **Metal levels, range/[IQR]** | **Association** | **Adjusted** | **Direction** |
| --- | --- | --- | --- | --- | --- | --- | --- | --- | --- | --- | --- | --- | --- | --- | --- | --- | --- |
| Manjarres-Suarez & Olivero-Verbel, 2020 | Cross-sectional | - | Colombia | Adolescents | All | - | 11-18 | Environmental (industrial areas) | Hair | Atomic absorption spectrometry | 194 | μg/g | 1.43 (0.07) | 0.04-4.81 | Spearman correlation: rho=-0162; p=0.024 | No | ▼ |

Supplementary Table 14. Summary of studies assessing the association between cadmium concentration and hemoglobin

| **Study** | **Design** | **Data collection timeframe** | **Country** | **Population** | **Sex included** | **Age, mean/ [median]** | **Age, range/ [IQR]** | **Source of exposure** | **Biological sample** | **Determination method** | **n (analysis)** | **Metal unit** | **Metal levels, mean (SD/SE)/ [median]** | **Metal levels, range/[IQR]** | **Association** | **Adjusted** | **Direction** |
| --- | --- | --- | --- | --- | --- | --- | --- | --- | --- | --- | --- | --- | --- | --- | --- | --- | --- |
| Kordas et al., 2010 | Cross-sectional | 2007 | Uruguay | Preschool children | All | 2 | 0-3 | - | Hair | Inductively coupled plasma mass spectrometry | 222 | μg/g | 0.28 (0.53) | <0.2-6.24 | Multivariate regression for log-transformed hair Cd levels: β=0.24 (0.20); p>0.1 | Yes - Age, sex, blood lead, maternal education, household possessions, and maternal hair metal level | ▲ |
| Nassef et al., 2014 | Cross-sectional | - | Egypt | School children with iron deficiency anemia | All | 10 | 6-12 | - | Blood | Graphite furnace atomic absorption spectrometry | 90 | μmol/L | Male: 0.11 (0.02)  Female: 0.10 (0.03) | - | Pearson correlation: r=-0.821; p<0.01 | No | ▼ |

Supplementary Table 15. Summary of studies assessing the association between arsenic concentration and hemoglobin

| **Study** | **Design** | **Data collection timeframe** | **Country** | **Population** | **Sex included** | **Age, mean/ [median]** | **Age, range/ [IQR]** | **Source of exposure** | **Biological sample** | **Determination method** | **n (analysis)** | **Metal unit** | **Metal levels, mean (SD/SE)/ [median]** | **Metal levels, range/[IQR]** | **Association** | **Adjusted** | **Direction** |
| --- | --- | --- | --- | --- | --- | --- | --- | --- | --- | --- | --- | --- | --- | --- | --- | --- | --- |
| Kordas et al., 2010 | Cross-sectional | 2007 | Uruguay | Preschool children | All | 2 | 0-3 | - | Hair | Inductively coupled plasma mass spectrometry | 222 | μg/g | 0.13 (0.14) | <0.014-1.00 | Multivariate regression for log-transformed hair As levels: β=0.51 (0.19); p<0.01 | Yes - Age, sex, blood lead, maternal education, household possessions, and maternal hair metal level | ▲ |
| López-Rodríguez et al., 2017 | Cross-sectional | 2010-2011 | Mexico | School children | All | Anemia: 9  No anemia: 10 | 6-12 | - | Venous blood | Scanning electron microscopy/energy-dispersive X-ray spectrometry | 40 | wt% | Anemia: 0.041 (0.11)  No anemia: 0.014 (0.05  p<0.05 | - | Spearman correlation: rho=-0.441; p<0.01 | No | ▼ |

**
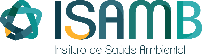

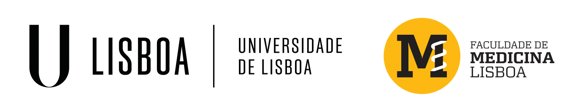
**
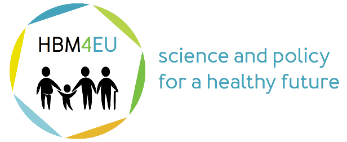


Exposure to heavy metals and red blood cells parameters in children: A systematic review of observational studies protocol

# Funding sources/sponsors

HBM4EU project is funded by the European Union’s Horizon 2020 Research and Innovation Programme under grant agreement No 733032.

# Conflicts of interest

The authors declare that they have no known conflicts of interest.

# Review question

Is the exposure to the HBM4EU prioritized heavy metals – lead (Pb), mercury (Hg), cadmium (Cd), arsenic (As), and chromium VI (Cr(VI)) – associated with changes in red blood cells (RBC) parameters in children?

# Aim

To summarize the available evidence of human studies exploring the association between exposure (internal dose) to the HBM4EU prioritized heavy metals – Pb, Hg, Cd, As, and Cr(VI) – and RBC parameters in children.

# Searches

Sources: PubMed, Scopus, and Web of Science electronic databases and eligible studies’ lists of references.

Search dates: from 1 January 2010 (to focus on the most up-to-date evidence available with more standardized methods to increase comparability between studies).

Restrictions: Peer-reviewed, published, full-text articles in English or Portuguese with empirical data.

Search will be re-run just before submission.

# Condition or domain being studied

RBC parameters.

# Participants/population

Inclusion: Children and adolescents aged 18 years or under.

Exclusion: Children with pre-specified health conditions not related to heavy metal exposure.

# Exposure(s)

Internal dose of HBM4EU prioritized heavy metals: Pb, Hg, Cd, As, and Cr(VI).

Studies with concomitant pharmacological interventions will be excluded.

# Comparator(s)/control

Not applicable.

# Types of study to be included

Study types included: Observational studies (cohort, case-control, cross-sectional).

Study types excluded: Pilot, feasibility, unfinished studies.

# Main outcome(s)

RBC parameters, including:

- Red blood cell count
- Hematocrit
- Hemoglobin
- Mean corpuscular volume
- Mean corpuscular hemoglobin
- Mean corpuscular hemoglobin concentration
- Red cell distribution width

## Measures of effect

Proportion of studies reporting a negative relationship between heavy metal levels and RBC parameters. The direction of the relationship will be assessed by mean/median differences between different heavy metal internal dose concentrations, correlation coefficients between the two variables, odds ratios for having higher levels of heavy metals or RBC parameters, and linear regression coefficients.

# Data extraction (selection and coding)

Microsoft Office Excel® will be used for the screening and extraction process.

After exclusion of duplicate records, all titles and abstracts of articles retrieved will be screened independently by the two authors according to the inclusion/exclusion criteria. Full-text copies of the eligible studies will be retrieved and will undergo a full-text review by two independent authors. Disagreements will be resolved by consensus or by a third independent reviewer.

Data will be extracted independently by two review authors, using a pre-piloted extraction form, which will include:

- General study information: author(s), year of publication, title.
- Study characteristics: study design, study years, population.
- Sample characteristics: sample size, age (mean/median and range), sex, country.
- Exposure characteristics: heavy metal, biological sample, determination method, unit, mean/median level, range, source of exposure.
- Outcome measures: red blood cell parameter, effect size/key findings, adjustments.

Disagreements will be resolved by consensus or by a third independent reviewer.

Study corresponding authors will be contacted to clarify and validate data extraction.

# Quality assessment

Studies quality will be assessed at the study level using the National Institutes of Health’s Study Quality Assessment Tools which assist reviewers in focusing on concepts that are key to a study’s internal validity. The tool used will depend on the study design: the Quality Assessment Tool for Observational Cohort and Cross-Sectional Studies and the Quality Assessment of Case-Control Studies.

Each study will be critically appraised by two authors independently. Studies will be classified as having good, fair, or poor quality. Discrepancies will be resolved by consensus or by a third author.

# Strategy for data synthesis

This systematic review will provide a quantitative synthesis of the available evidence on the association between each heavy metal internal dose and each RBC parameter, using vote counting based on the direction of the relationship between the two variables.

This method compares the number of studies indicating negative relationships with the number of studies indicating positive relationships regarding a specific outcome, independently of the reported *p* value. An estimate of the proportion (*p*) of studies reporting negative relationships will be calculated as *p* = *u*/*n*, where *u* = number of negative relationships, and *n* = number of studies, along with a 95% confidence interval, using Jeffreys interval methods. Besides a positive or negative relationship, studies may also be considered to have conflicting results or to have no direction. We will consider conflicting results when a study reports relationships in opposite directions between subgroups (e.g., male/female, exposed vs. control group) or between different biological samples (e.g., blood/erythrocyte). We will consider having no direction when a relationship has no direction (e.g., when the differences between means or the correlation coefficient equal zero).

# Analysis of subgroups or subsets

As sensitivity analysis, we will estimate the proportion of studies reporting negative relationships excluding the studies with conflicting results and excluding studies rated as having fair or poor quality.
